# Supplementary material for: EUROlinkCAT protocol for a European population-based data linkage study investigating the survival, morbidity and education of children with congenital anomalies
Source: BMJ Open. 2021 Jun 28;11(6):e047859. doi: 10.1136/bmjopen-2020-047859 (PMC8240574; doi:10.1136/bmjopen-2020-047859)
Supplement: Supplementary data [file bmjopen-2020-047859supp001.pdf]

Variables in EUROlinkCAT Common Data Model: Mortality

Variable Name	***Variable Definition and Instructions***	***Variable Format***	***Variable Values***
**Core variable required for linkage to vital statistics or mortality databases and for calculating age at death**			
L_CH_ID	Unique identifier of child<br><br>A unique ID that links child to another database	As recorded locally	
L_CH_DATE_B	Child's date of birth<br><br>Needed to calculate age at death.	DDMonYYYY	
L_CH_DATE_D	Child's date of death<br><br>Needed to calculate age at death.	DDMonYYYY	
**Variables relating to linkage**			
L_MATCH_TYPE	Match with vital statistics database (or local health care databases)	Numeric	1 = Linked to national/vital statistics database – match<br>2 = Linked to national/vital statistics database – non-match<br>3 = Linked to mortality database only – match<br>4 = Linked to mortality database only – non-match<br>5 = EUROCAT death only

Morris JK, *et al. BMJ Open* 2021; 11:e047859. doi: 10.1136/bmjopen-2020-047859

Variable Name	***Variable Definition and Instructions***	***Variable Format***	***Variable Values***
L_CONFIDENCE	Strength of match with vital statistics or health care database.<br><br>Use local data provider's codes for assessing confidence that the case is correctly matched. If local code unavailable, use suggested coding (see Appendix for full details)	Numeric	1=Excellent<br>2=Good<br>3=Fair<br>4=Poor<br>9=Not Matched
L_DATE_LOST	Date lost to follow-up/ linkage (i.e. due to emigration, adoption or other reason)	DDMonYYYY	.=Not recorded by registry or not available for study
L_YEAR_LOST	Year lost to follow-up/ linkage (i.e. due to emigration, adoption or other reason)	YYYY	.=Not recorded by registry or not available for study
L_AGEL_D	Age lost to follow-up/ linkage <u>in complete days</u>	Numeric (1-4 digits)	.=Not recorded by registry or not available for study
**Standardised variables relating to child**			
L_CH_YEAR_B	Child's year of birth	YYYY	
L_CH_SEX	Child's sex	Numeric	1 = Male<br>2 = Female<br>3=Indeterminate<br>9 = Not known<br>.=Not recorded by registry or not available for study
L_CH_REG_TYPE	Type of birth/ civil registration of baby	Numeric	1 = Livebirth<br>2 = Stillbirth<br>9 = Not known<br>.=Not recorded by registry or not available for study
L_CH_NON_EUR	Citizenship/ Nationality of infant /country of origin	Numeric	1= National<br>2= Other European<br>3 = Non-European

Variable Name	Variable Definition and Instructions	Variable Format	Variable Values
			4= Non-national (exact nationality not specified)<br>9 = Not known<br>. = Not recorded by registry or not available for study
L_CH_BW	Child's birthweight (grams)	Numeric	9999=Not known<br>.=Not recorded by registry or not available for study
L_CH_GA_B	Child's gestational age at birth (completed weeks)	Numeric	99=Not known<br>.=Not recorded by registry or not available for study
**Variables relating to mortality**			
L_CH_STATUS	Outcome status<br>-Died = child is known to have died before 10th birthday or 31 Dec 2015 (whichever earlier)<br><br>-Alive at 10th birthday = child was born on or before the 31st Dec 2005 and:<br><br>(i) is definitively known to be alive on 10th birthday; or (ii) there is no information on death or lost to follow-up<br><br>- Censored on 31st Dec 2015 = child was born on or after 1st Jan 2006 and:<br><br>(i) is definitively known to be alive on 31st Dec 2015; or (ii) there is no information on death or lost to follow-up<br><br>-Lost to follow up = child is lost to follow-up/ linkage (i.e. due to emigration, adoption or other reason)	Numeric	1 = Died<br>2 = Alive at 10th birthday<br>3 = Censored on 31st Dec 2015<br>4 = Lost to follow up

Variable Name	***Variable Definition and Instructions***	***Variable Format***	***Variable Values***
L_EXIT_DATE	Date of last day in study (censored/lost/died/alive)<br><br>- Date lost - if child was lost to follow up<br><br>- Date of death - if child has died<br><br>- Date of birth plus 3652 days (approx. 10th birthday) if child was born on or before the 31st Dec 2005 and:<br><br>(i) is definitively known to be alive on 10th birthday; or (ii) there is no information on death or lost to follow-up<br><br>- Date of last day of the study (31st Dec 2015) if child was born on or after 1st Jan 2006 and:<br><br>(i) is definitively known to be alive on 31st Dec 2015; or (ii) there is no information on death or lost to follow-up	DDMonYYYY	
L_EXIT_DAYS	Number of days child is in study<br><br>This is calculated as the last date child was in the study (L_EXIT_DATE) minus the child's birth date (L_CH_DATE_B).	Numeric (1-4 digits)	
L_CH_YEAR_D	Year of child's death as recorded on the death certificate	YYYY	
L_CH_AGED_H	Age at death in complete hours for day 0 (first 24 hours) = applies to infants who died within the first 24 hours<br><br>This variable can be used to check values recorded under variable L_CH_AGED_D.<br><br>A child who died within 24 hours but across two dates is coded "0" on L_CH_AGED_D below.	Numeric<br><br>(1-2 digits)	0 = Died <1 hour after birth<br>1 = Died 1 complete hour after birth<br>2 = Died 2 complete hours after birth<br>Etc.<br>23 = Died 23 complete hours after birth<br>88 = Alive at 24 hours<br>99 = Died within first 24 hours, but exact time unknown

Morris JK, *et al. BMJ Open* 2021; 11:e047859. doi: 10.1136/bmjopen-2020-047859

Variable Name	***Variable Definition and Instructions***	***Variable Format***	***Variable Values***
L_CH_AGED_D	Age at death in complete days (up to 10th birthday).<br><br>A calculated field using the difference between date of death and death of birth i.e. subtract child's date of birth from child's date of death.	Numeric (1-4 digits)	0 = died <24 hours after birth<br>1 = died 1 complete day after birth<br>2 = died 2 complete days after birth<br>Etc.<br>8888 = Alive on 10th birthday or by end of study period, whichever is sooner<br>9999 = Died before 10th birthday, but exact time unknown
L_CAUSE_D_U	Underlying cause of death - diagnosis<br><br>There can only be one underlying cause of death	String	ICD9 or ICD10 code
L_CAUSE_D_P	Primary/ immediate cause of death - diagnosis	String	ICD9 or ICD10 code
L_CAUSE_D_C	Contributing cause of death - diagnosis	String	ICD9 or ICD10 code
L_CAUSE_D_O1	Other causes of death- diagnosis 1	String	ICD9 or ICD10 code
L_CAUSE_D_O2	Other causes of death- diagnosis 2	String	ICD9 or ICD10 code
L_CAUSE_D_O3	Other causes of death- diagnosis 3	String	ICD9 or ICD10 code
L_CAUSE_D_O4	Other causes of death- diagnosis 4	String	ICD9 or ICD10 code
L_CAUSE_D_O5	Other causes of death- diagnosis 5<br><br>Additional "Other causes of death" can be recorded in variables L_CAUSE_D_O6 to L_CAUSE_D_15 (not listed)	String	ICD9 or ICD10 code
L_CH_PLACE_D	Place of death	Numeric	1 = Home<br>2 = Hospital<br>3 = Other<br>9 = Not known<br>.=Not recorded by registry or not available for study

Morris JK, *et al. BMJ Open* 2021; 11:e047859. doi: 10.1136/bmjopen-2020-047859

Variable Name	*Variable Definition and Instructions*	*Variable Format*	*Variable Values*
**Variables relating to mother**			
L_MAT_YEAR_B	Maternal year of birth	YYYY	99 = Not known<br><br>. = Not recorded by registry or not available for study
L_MATAGE_B	Maternal age at infant's birth in completed years<br><br>May be used to link information held on the child in local health care databases	Numeric	99=Not known<br>. =Not recorded by registry or not available for study
L_MULT_BIRTH	Singleton or multiple birth	Numeric	1=Singleton<br>2=Twins<br>3=Triplets or higher<br>4= Multiple birth, number unknown<br>9= Not known<br>. =Not recorded by registry or not available for study
L_MAT_CTRY_B	Maternal country of birth/ place of birth/ country of origin	Numeric	1= National<br>2= Other European<br>3 = Non-European<br>4= Non-national (exact nationality not specified)<br>9 = Not known<br>.=Not recorded by registry or not available for study
L_MAT_BMI	Maternal Body Mass Index (BMI) at first antenatal visit/at booking<br><br>Expected range 15 – 50	Numeric<br><br>(Whole number only)	Exact BMI value<br>97 = <30<br>98 = >=30<br>99 = Not known<br>.=Not recorded by registry or not available for study
L_MAT_EDUC	Maternal education	Numeric	1 = Pre-primary /Primary<br>2 = Any secondary<br>3 = Postsecondary (non-tertiary)

Variable Name	***Variable Definition and Instructions***	***Variable Format***	***Variable Values***
	(UNESCO's International Standard Classification of Education (ISCED))		4 = Tertiary<br>5=No education<br>9 = Not known<br>. = Not recorded by registry or not available for study
L_MAT_OCC	Maternal occupation<br><br>(Based on Paris (INSERM) coding classification)	Numeric	1= Farmer<br>2= Artisan (ex: baker) / shop-owner<br>3= Professional<br>4= Intermediate<br>5= Administrative/public service<br>6= Business employees: shop assistants/ salesperson<br>7= Household and personal service<br>8= Skilled manual worker<br>9=Unskilled manual worker<br><br>0= No occupation declared / student<br>99= Not known<br>. =Not recorded by registry or not available for study
L_MATDEPR_IND	Deprivation index at maternal residence<br><br>Multiple deprivations scores are ranked into quintiles where 1= Least deprived and 5 = Most deprived (coding scheme used in Wales & Basque Country)	Numeric	1 = First quintile (Least deprived)<br>2 = Second quintile<br>3 = Third quintile<br>4 = Fourth quintile<br>5 = Fifth quintile (Most deprived)<br>9 = Not known<br>. =Not recorded by registry or not available for study
L_PROXY_SES	Proxy variable for SES<br><br>This is registry-specific. Use the agreed proxy variable for each registry.<br><br>Maternal education:<br>• Tertiary/ post-secondary=High<br>• Any secondary = Middle	Numeric	1 = High<br>2 = Middle<br>3 = Low<br>9=Not known<br>. = Not recorded by registry or not available for study

Variable Name	***Variable Definition and Instructions***	***Variable Format***	***Variable Values***
	• Primary/ pre-primary/ No education = Low<br><br>Maternal occupation:<br>• Professional = High<br>• Intermediate= Middle<br>• No occupation = Low<br><br>Multiple Deprivation Index<br>• Quintile 1 (Least deprived)=High<br>• Quintiles 2-4= Middle<br>• Quintile 5 (Most deprived)= Low		
L_MATMAR_STA	Maternal marital status	Numeric	1 = Single<br>2 = Married/ Living together<br>3 = Widow<br>4 = Divorced/ Separated<br>9 = Not known<br>. =Not recorded by registry or not available for study
**Recoded variables**			
Yeargp	Grouped year of birth<br>1995/2004=1<br>2005/2014 =2	Numeric	1 = 1995-2004<br>2 = 2005-2014
BMI_gp	Grouped BMI<br><br>BMI <10, code as unknown<br>BMI >60, code as unknown<br>Blank or missing, code as unknown	Numeric	1 = <30<br>2 = 30+<br>9 = unknown
BW_gp	Grouped Birthweight<br><br>BW <400g, code as unknown<br>BW >7000g, code as unknown<br>Blank or missing, code as unknown	Numeric	1= <1000g<br>2 = 1000-1499g<br>3 = 1500-2499g<br>4 = 2500-3999g<br>5 = 4000+ g<br>9 = unknown
GA_gp	Grouped Gestational age	Numeric	1 = 24-27weeks<br>2 = 28-31 weeks

Morris JK, *et al. BMJ Open* 2021; 11:e047859. doi: 10.1136/bmjopen-2020-047859

Variable Name	***Variable Definition and Instructions***	***Variable Format***	***Variable Values***
	GA <24 weeks, excluded from study<br>GA >45 weeks, code as unknown<br>Blank or missing, code as unknown		3= 32-36 weeks<br>4 = 37+ weeks<br>9 = unknown
GA_disc_gp	Grouped Gestational age at discovery<br><br>GA at discovery <8 weeks, code as unknown<br>GA at discovery >42 weeks, code as unknown<br>Blank or missing, code as unknown	Numeric	1= <22 weeks<br>2 = 22-31 weeks<br>3 = 32+ weeks<br>9 = unknown
matage_gp	Grouped Maternal age at infant's birth<br><br>Maternal age range<br>10-19 years, code =1<br>20-34 years, code=2<br>35-59 years, code=3<br>All other values, blanks or missing, code=9	Numeric	1= <20 years<br>2= 20-34 years<br>3= 35+ years<br>9=Not known

Morris JK, *et al. BMJ Open* 2021; 11:e047859. doi: 10.1136/bmjopen-2020-047859
