# Supplementary material for: EUROlinkCAT protocol for a European population-based data linkage study investigating the survival, morbidity and education of children with congenital anomalies
Source: BMJ Open. 2021 Jun 28;11(6):e047859. doi: 10.1136/bmjopen-2020-047859 (PMC8240574; doi:10.1136/bmjopen-2020-047859)
Supplement: Supplementary data [file bmjopen-2020-047859supp002.pdf]

Variables in EUROlinkCAT Common Data Model: Health Care

Variable name	***Variable definition/ description***	***Variable format***	***Variable values***	***Tables***[1]
**Table: Patient level data**<br>***One row per child***<br>***Core variables for linkage to hospital discharge records and for calculating age at event e.g. hospitalisation***				
L_CH_ID	Unique identifier of child<br><br>A unique ID that links child to another database	As recorded locally		- Patient<br>- EUROCAT case data
L_CH_REC	Unique child record number<br><br>Generated by syntax script for all children (cases and controls), irrespective of whether the child has been in hospital<br><br>For children with a hospital admission, this is used to link admission, diagnoses, procedures & surgeries and prescriptions to child	Numeric		- Patient<br>- Hospital admission<br>- Diagnoses<br>- Procedures & surgeries<br>- Prescription
PROVIDER_ID	Unique reference number as present in the provider's linked data	String		- Patient<br>- Hospital admission<br>- Diagnoses<br>- Procedures & surgeries<br>- Prescription
L_CASECON	Case or control status (apply status AFTER linkage is complete)<br><br>Exclusions:<br>- child with gestational age <23 weeks<br><br>Control with ICD10 Q code or ICD9 (-CM) 740-759 codes recorded. This is a child with a congenital anomaly code (ICD10 Q code, or ICD9-CM 740-759) in hospital discharge records that is not recorded as a EUROCAT case.<br><br>Children coded as 1 or 2 will be included in WP4 analysis.<br><br>Children coded as 3 are required for WP6	Numeric	1= Case<br>2= Control with no ICD10 Q code or ICD9(-CM) 740-759 codes recorded<br>3=Control with ICD10 Q code or ICD9(-CM) 740-759 codes recorded	- Patient<br>- Hospital admission<br>- Diagnoses<br>- Procedures & surgeries<br>- Prescription
L_HOSP_ADM	Admitted to hospital (up to 10th birthday)<br><br>Exclude obstetric stays i.e. hospital stays relating to the birth and Accident & Emergency/ Emergency room stays	Binary	0= No hospital admissions<br>1= One or more hospital admission (s)	- Patient

Variable name	***Variable definition/ description***	***Variable format***	***Variable values***	***Tables[1]***
L_PRESCRIPT	Prescription for one of the 5 specified conditions*:* Asthma, Cardiac, Diabetes, Epilepsy, and Infections.<br><br>N.B. UK prescriptions are issued by a GP, while in continental Europe, prescriptions are dispensed by the pharmacy (either community or hospital outpatient pharmacy)	Numeric	0= No prescription<br>1= One or more prescription (s)	- Patient
L_CH_DATE_B	Child's date of birth<br><br>Used to calculate age at hospitalisation, diagnoses, procedures, & prescriptions	DDMonYYYY		- Patient
L_CH_YEAR_B	Child's year of birth	YYYY		- Patient
L_CH_DATE_D	Child's date of death	DDMonYYYY		- Patient
L_CH_YEAR_D	Child's year of death	YYYY		- Patient
L_CH_AGED_D	Age at death in complete days (up to 10th birthday).<br><br>A calculated field using the difference between date of death and death of birth i.e. subtract child's date of birth from child's date of death.	Numeric (1-4 digits)	0 = died <24 hours after birth<br>1 = died 1 complete day after birth<br>2 = died 2 complete days after birth<br>Etc.<br>8888 = Alive on 10th birthday or by end of study period, whichever is sooner<br>9999 = Died before 10th birthday, but exact time unknown	- Patient
**Information on risk factors – needed for all children**				
L_MATAGE_B	Maternal age at infant's birth in completed years	Numeric	99 = Not known<br>. = Not recorded or not available for study	- Patient
L_MATMAR_STA	Maternal marital status at delivery	Numeric	1 = Single<br>2 = Married/ Living together<br>3 = Widow<br>4 = Divorced/ Separated<br>9 = Not known<br>. = Not recorded or not available for study	- Patient
L_MAT_CTRY_B	Maternal country of birth/ place of birth/ country of origin	Numeric	1= National<br>2= Other European<br>3 = Non-European<br>4= Non-national (exact nationality not specified)<br>9 = Not known<br>. = Not recorded or not available for study	- Patient
L_MAT_BMI	Maternal Body Mass Index (BMI) at first antenatal visit/at booking<br><br>Expected range 15 – 50	Numeric<br><br>(Whole number only)	Exact BMI value<br>97 = <30<br>98 = >=30<br>99 = Not known<br>. = Not recorded or not available for study	- Patient

Variable name	*Variable definition/ description*	*Variable format*	*Variable values*	*Tables[1]*
L_MAT_EDUC	Maternal education<br><br>(UNESCO's International Standard Classification of Education (ISCED))	Numeric	1 = Pre-primary /Primary<br>2 = Any secondary<br>3 = Postsecondary (non tertiary)<br>4 = Tertiary<br>5=No education<br>9 = Not known<br>. = Not recorded or not available for study	- Patient
L_MATDEPR_IND	Deprivation index at maternal residence<br><br>Multiple deprivations scores are ranked into quintiles where 1= Least deprived and 5 = Most deprived (coding scheme used in Wales & Basque Country)	Numeric	1 = First quintile (Least deprived)<br>2 = Second quintile<br>3 = Third quintile<br>4 = Fourth quintile<br>5 = Fifth quintile (Most deprived)<br>9 = Not known<br>. = Not recorded or not available for study	- Patient
L_PROXY_SES	Proxy variable for Socio-Economic Status (SES)<br><br>This is registry-specific. Use the agreed proxy variable for each registry.<br><br><u>Maternal education</u>:<br>• Tertiary/ post-secondary=High<br>• Any secondary = Middle<br>• Primary/ pre-primary/ No education = Low<br><br><u>Maternal occupation:</u><br>• Professional = High<br>• Intermediate= Middle<br>• No occupation = Low<br><br><u>Multiple Deprivation Index</u><br>• Quintile 1 (Least deprived) =High<br>• Quintiles 2-4= Middle<br>• Quintile 5 (Most deprived) = Low	Numeric	1 = High<br>2 = Middle<br>3 = Low<br>9=Not known<br>. = Not recorded or not available for study	- Patient
L_MULT_BIRTH	Singleton or multiple birth	Numeric	1=Singleton<br>2=Twins<br>3=Triplets or higher<br>4= Multiple birth, number unknown<br>9= Not known<br>. = Not recorded or not available for study	- Patient
L_CH_SEX	Child's sex	Numeric	1 = Male<br>2 = Female<br>3 = Indeterminate<br>9 = Not known<br>. = Not recorded or not available for study	- Patient
L_CH_BW	Child's birth weight (grams)	Numeric	9999 = Not known<br>. = Not recorded or not available for study	- Patient

Variable name	Variable definition/ description	Variable format	Variable values	Tables[1]
L_CH_GA_B	Child's gestational age at birth (in completed weeks).	Numeric	99 = Not known<br>. = Not recorded or not available for study	- Patient
L_PRENATAL	Prenatal diagnosis of congenital anomaly<br><br>[EUROCAT case only, i.e. L_CASECON=1 All control children in the linked dataset (i.e. L_CASECON=2 or L_CASECON=3) should be coded as .]<br><br>[Derived from the EUROCAT variables when discovered (WHENDISC) and age at prenatal diagnosis (AGEDISC)]	Numeric	0 = Not prenatally diagnosed<br>1 = Prenatally diagnosed, <22 weeks<br>2 = Prenatally diagnosed, 22-31 weeks<br>3 = Prenatally diagnosed, 32+ weeks<br>4 = Prenatally diagnosed, GA unknown<br>9 = Not known if prenatally diagnosed<br>.= Not recorded or not available for study	-Patient
**Recoded variables**				
Yeargp_WP4	Grouped year of birth<br><br>1995/2004=1<br>2005/2009 =2<br>2010-2014 = 3	Numeric	1 = 1995-2004<br>2 = 2005-2009<br>3 = 2010 - 2014	-Patient
Yeargp_Px	Grouped year of birth for the prescription study<br><br>2000/2004=1<br>2005/2009 =2<br>2010-2014 = 3	Numeric	1 = 2000-2004<br>2 = 2005-2009<br>3 = 2010 - 2014	-Patient
BMI_gp	Grouped BMI<br><br>BMI <10, code as unknown<br>BMI >60, code as unknown<br>Blank or missing, code as unknown	Numeric	1 = <30<br>2 = 30+<br>9 = unknown	-Patient
BW_WP4_gp	Grouped Birth weight (BW)<br><br>BW <400g, code as unknown<br>BW >7000g, code as unknown<br>Blank or missing, code as unknown	Numeric	1= <1500g<br>2 = 1500-2499g<br>3 = 2500-3999g<br>4 = 4000+ g<br>9 = unknown	-Patient
GA_WP4_gp	Grouped Gestational age<br><br>GA <23 weeks, excluded from study<br>GA >44 weeks, code as unknown<br>Blank or missing, code as unknown	Numeric	1 = 23-27 weeks<br>2 = 28-31 weeks<br>3 = 32-36 weeks<br>4 = 37+ weeks<br>9 = unknown	-Patient
GA_disc_gp	Grouped Gestational age at discovery<br><br>GA at discovery <8 weeks, code as unknown<br>GA at discovery >42 weeks, code as unknown<br>Blank or missing, code as unknown	Numeric	1= <22 weeks<br>2 = 22-31 weeks<br>3 = 32+ weeks<br>9 = unknown	-Patient
Matage_gp	Grouped Maternal age at infant's birth<br><br>Maternal age range<br>10-19 years, code =1<br>20-34 years, code=2<br>35-59 years, code=3<br>All other values, blanks or missing, code=9	Numeric	1= <20 years<br>2= 20-34 years<br>3= 35+ years<br>9=Not known	-Patient

Variable name	*Variable definition/ description*	*Variable format*	*Variable values*	*Tables[1]*
Derived variables relating to linkage				
L_MATCH_TYPE_V	Match with national/ vital statistics database	Numeric	1 = Linkage to national/vital statistics database - match<br>2 = Linkage to national/vital statistics database - non-match<br>3 = EUROCAT death only<br>. = No linkage to national/ vital statistics	-Patient
L_MATCH_TYPE_H	Match with hospital database<br><br>The value 4 "Only matched to hospital discharge database outside the study period or matched to hospital outpatient records in the study period" is included for matching purposes only i.e. the hospital stay for this child is not included in analysis.<br><br>The rationale is that if a child is not matched to hospital discharge records during the study period, but is found in hospital records AFTER the study period, then we can be more confident that the child did not have a hospital admission during our study period.<br><br>Similarly, if a child is found in outpatient records during the study period but is not found in the hospital discharge records, we can be more confident that the child did not have a hospital admission during our study period.	Numeric	1 = Linkage to hospital database - match<br>2 = Linkage to hospital database - non-match<br>3 = EUROCAT death only<br>4 = Only matched to hospital discharge database outside the study period or matched to hospital outpatient records in the study period	-Patient
L_CONFID_HDR	Strength of match with hospital database.<br><br>Use local data provider's codes for assessing confidence that the case is correctly matched. If local code unavailable, use suggested coding<br><br>The value 0 represents a child:<br>• not matched to hospital discharge records in the study period, but found in other databases within national statistics (such as outpatient records)<br>• found only in hospital discharge records AFTER the study period.	Numeric	0=Found in other database or found only in hospital database outside the study period<br>1=Excellent<br>2=Good<br>3=Fair<br>4=Poor<br>9=Not Matched	- Patient
L_MORB_INCLUDE	Include child in WP4 analysis<br><br>Used in sensitivity analysis to assess if a child should be included in the WP4 analysis or not i.e. it will assess confidence that a child definitely did not go to hospital if hospital admission (variable L_HOSP_ADM = 0).<br><br>Please see appendix 1 for algorithm)	Binary	0 = Do not include in analysis<br>1 = Include in analysis	- Patient

Variable name	*Variable definition/ description*	*Variable format*	*Variable values*	*Tables[1]*
L_MATCH_PX	Match with GP / community pharmacy prescription database / hospital outpatient pharmacy records	Numeric	1 = Linked prescription database – match<br>2 = Linked to prescription database – non-match<br>3 = Linkage to prescription data not available	- Patient
L_CONFID_PX	Strength of match with prescription database.<br><br>The value 9 represents a child not matched/ found in the local GP/ pharmacy prescription records (i.e. L_MATCH_PX=2);<br>or a child whose GP/ pharmacy do not contribute data to this study (i.e. L_MATCH_PX=3).<br><br>Children coded as L_MATCH_PX=3 will be excluded from analysis.	Numeric	1=Excellent<br>2=Good<br>3=Fair<br>4=Poor<br>9=Not Matched	- Patient
L_DATE_LOST	Date lost to follow-up/ linkage (i.e. due to emigration, adoption or other reason)	DDMonYYYY	. = Not recorded or not available for study	- Patient
L_YEAR_LOST	Year lost to follow-up/ linkage (i.e. due to emigration, adoption or other reason)	YYYY	. = Not recorded or not available for study	- Patient
L_AGEL_D	Age lost to follow-up/ linkage <u>in complete days</u>	Numeric (1-4 digits)	. = Not recorded or not available for study	- Patient
L_EXIT_DATE	Date of last day in study (censored/lost/died/alive)<br><br>- Date lost - if child was lost to follow up<br>- Date of death - if child has died<br>- Date of birth plus 3652 days (approx. 10th birthday) if child was born on or before the 31st Dec 2005 and:<br>(i) is definitively known to be alive on 10th birthday; or (ii) there is no information on death or lost to follow-up<br>- Date of last day of the study (31st Dec 2015) if child was born on or after 1st Jan 2006 and:<br>(i) is definitively known to be alive on 31st Dec 2015; or (ii) there is no information on death or lost to follow-up	DDMonYYYY		- Patient
L_EXIT_DAYS	Number of days child is in study.<br><br>This is calculated as the last date child was in the study (L_EXIT_DATE) minus the child's birth date (L_CH_DATE_B).	Numeric (1-4 digits)		- Patient
L_CH_STATUS	Outcome status<br><br>-Died = child is known to have died before 10th birthday or 31 Dec 2015 (whichever earlier)	Numeric	1 = Died<br>2 = Alive at 10th birthday<br>3 = Censored on 31st Dec 2015<br>4 = Lost to follow up	- Patient

Variable name	Variable definition/ description	Variable format	Variable values	Tables[1]
	-Alive at 10th birthday = child was born on or before the 31st Dec 2005 and:<br>(i) is definitively known to be alive on 10th birthday; or (ii) there is no information on death or lost to follow-up<br><br>- Censored on 31st Dec 2015 = child was born on or after 1st Jan 2006 and:<br>(i) is definitively known to be alive on 31st Dec 2015; or (ii) there is no information on death or lost to follow-up<br><br>-Lost to follow up = child is lost to follow-up/ linkage (i.e. due to emigration, adoption or other reason)			
**Table: Hospital admission variables**<br>***(excluding Obstetric stays and Accident & Emergency/ Emergency room stays)***<br>***One row for each hospital admission.***<br>***If multiple hospital admissions, please complete all hospital admission variables for each admission***				
L_CH_REC	Unique child record number<br><br>Generated by syntax script for all children (cases and controls), irrespective of whether the child has been in hospital<br><br>For children with a hospital admission, this is used to link admission, diagnoses, procedures & surgeries and prescriptions to child	Numeric		- Patient<br>- Hospital admission<br>- Diagnoses<br>- Procedures & surgeries<br>- Prescription
PROVIDER_ID	Unique reference number as present in the provider's linked data	String		- Patient<br>- Hospital admission<br>- Diagnoses<br>- Procedures & surgeries<br>- Prescription
L_ADM_REC	Unique admission record number<br><br>Used to link the child's admission records to the child's diagnoses and procedures & surgeries during that admission<br><br>Generated by syntax script	Numeric		- Hospital admission - Diagnoses<br>- Procedures & surgeries
L_CASECON	Case or control status (apply status AFTER linkage is complete)<br><br>Exclusions:<br>- child with gestational age <23 weeks<br><br>Control with ICD10 Q code or ICD9 (-CM) 740-759 codes recorded. This is a child with a congenital anomaly code (ICD10 Q code, or ICD9-CM 740-759) in hospital discharge records that is not recorded as a EUROCAT case.<br><br>Children coded as 1 or 2 will be included in WP4 analysis.<br>Children coded as 3 are required for WP6	Numeric	1= Case<br>2= Control with no ICD10 Q code or ICD9(-CM) 740-759 codes recorded<br>3=Control with ICD10 Q code or ICD9(-CM) 740-759 codes recorded	- Patient<br>- Hospital admission<br>- Diagnoses<br>- Procedures & surgeries<br>- Prescription

Variable name	***Variable definition/ description***	***Variable format***	***Variable values***	***Tables[1]***
L_HOSP_SEQ	**<u>This variable is only required for the registries: registry numbers 13, 28</u>**<br><br>Sequential number allocated to each hospital in registry area e.g. Hospital A=1, Hospital B=2 etc.<br><br>Purpose of this variable is to identify the hospital for each record. It is known that some hospitals in some registries are missing >20% hospital admissions in specific years. This variable will be used in sensitivity analysis, to include/ exclude the hospital (s) with >20% missing information to see its effect on the analysis.	Numeric	. = Not recorded or not available for study	- Hospital admission
L_DATE_ADM	Date of admission to hospital	DDMonYYYY		- Hospital admission
L_YEAR_ADM	Year of admission to hospital	YYYY		- Hospital admission
L_DATE_DIS	Date of discharge from hospital	DDMonYYYY		- Hospital admission
L_YEAR_DIS	Year of discharge from hospital	YYYY		- Hospital admission
L_HOSP_DAYS	Length of stay in hospital in days<br><br>Subtract discharge date from hospital admission date.<br><br>If value =0 for children admitted and discharged on the same day, replace value 0 with value 0.5	Numeric	0.5 = <1 day<br>1=1 day/ over-night stay<br>2=2 days etc<br>999= Not known	- Hospital admission
L_AGE_ADM	Child's age at hospital admission <u>in days</u> (up to 10th birthday).<br><br>Subtract child's date of birth from date of admission to hospital	Numeric	0= <1 day old<br>1= 1 day old<br>2= 2 days old etc<br>9999= exact age not known	- Hospital admission
L_ICU_ADM	Admission to Neonatal, Paediatric or other Intensive Care Unit (ICU) during this hospital admission	Numeric	0= No ICU admission<br>1=ICU admission<br>9 = Not known<br><br>. = Not recorded or not available for study	- Hospital admission
L_VENT	Mechanical ventilator use during this hospital admission<br><br>Non-invasive ventilation is excluded	Numeric	0= No<br>1= Yes<br>9= Not known<br>. = Not recorded or not available for study	- Hospital admission

Table: Diagnosis variables (up to 10th birthday)

Each diagnosis is a separate observation (row) in the table
<u>0, 1 or more rows for each hospital admission</u>
If multiple diagnoses, please provide date of each diagnosis

L_CH_REC	Unique child record number	Numeric		- Patient<br>- Hospital admission<br>- Diagnoses

Variable name	*Variable definition/ description*	*Variable format*	*Variable values*	*Tables*[1]
	Generated by syntax script for all children (cases and controls), irrespective of whether the child has been in hospital<br><br>For children with a hospital admission, this is used to link admission, diagnoses, procedures & surgeries and prescriptions to child			- Procedures & surgeries<br>- Prescription
PROVIDER_ID	Unique reference number as present in the provider's linked data	String		- Patient<br>- Hospital admission<br>- Diagnoses<br>- Procedures & surgeries<br>- Prescription
L_ADM_REC	Unique admission record number<br><br>Used to link the child's admission records to the child's diagnoses and procedures during that admission<br><br>Generated by syntax script	Numeric		- Hospital admission<br>- Diagnoses<br>- Procedures & surgeries
L_CASECON	Case or control status (apply status AFTER linkage is complete)<br><br>Exclusions:<br>- child with gestational age <23 weeks<br><br>Control with ICD10 Q code or ICD9 (-CM) 740-759 codes recorded. This is a child with a congenital anomaly code (ICD10 Q code, or ICD9-CM 740-759) in hospital discharge records that is not recorded as a EUROCAT case.<br><br>Children coded as 1 or 2 will be included in WP4 analysis.<br><br>Children coded as 3 are required for WP6	Numeric	1= Case<br>2= Control with no ICD10 Q code or ICD9(-CM) 740-759 codes recorded<br>3=Control with ICD10 Q code or ICD9(-CM) 740-759 codes recorded	- Patient<br>- Hospital admission<br>- Diagnoses<br>- Procedures & surgeries<br>- Prescription
L_DIAG	Diagnosis in ICD9 or ICD10 for the hospital stay	String<br><br>As recorded in the hospital database		- Diagnoses
L_DATE_DIAG	Date of diagnosis<br><br>Use date of discharge as a proxy for date of diagnosis, if date of diagnosis is not available	DDMonYYYY		- Diagnoses
L_AGE_DIAG	Child's age at diagnosis in days (up to 10th birthday).<br><br>Subtract child's date of birth from date of diagnosis to calculate the child's age at diagnosis. If date of diagnosis is not available, then use date of discharge	Numeric	0= <1 day old<br>1= 1 day old<br>2= 2 days old etc<br>9999= exact age not known	- Diagnoses

Morris JK, *et al. BMJ Open* 2021; 11:e047859. doi: 10.1136/bmjopen-2020-047859

Variable name	***Variable definition/ description***	***Variable format***	***Variable values***	***Tables[1]***
L_CANCER	A diagnosis of cancer (ICD9 or ICD10)	Binary	0 = No<br>1 = Yes	- Diagnoses
L_CPALSY	A diagnosis of cerebral palsy (ICD9 or ICD10)	Binary	0 = No<br>1 = Yes	- Diagnoses
L_EPILEPSY	A diagnosis of epilepsy or seizures (ICD9 or ICD10)	Binary	0 = No<br>1 = Yes	- Diagnoses
L_RENAL	A diagnosis of renal failure (ICD9 or ICD10)	Binary	0 = No<br>1 = Yes	- Diagnoses
L_HEARING	A diagnosis of hearing loss (ICD9 or ICD10)	Binary	0 = No<br>1 = Yes	- Diagnoses
L_VISION	A diagnosis of visual impairment or blindness (ICD9 or ICD10)	Binary	0 = No<br>1 = Yes	- Diagnoses
L_INJURYPOISON	A diagnosis of injury or poisoning (ICD9 or ICD10)	Binary	0 = No<br>1 = Yes	- Diagnoses
L_BATTERED	A diagnosis of physical abuse (battered child) (ICD9 or ICD10)	Binary	0 = No<br>1 = Yes	- Diagnoses
**Table: Surgery and Procedures (including admission to intensive care unit)**<br>***Each surgery/ procedure /ICU admission is a separate observation (row) in the table***<br>***0,1 or more rows for each hospital admission***<br>***If multiple procedures, please provide procedure date and age of child at each procedure***				
L_CH_REC	Unique child record number<br><br>Generated by syntax script for all children (cases and controls), irrespective of whether the child has been in hospital<br><br>For children with a hospital admission, this is used to link admission, diagnoses, procedures & surgeries and prescriptions to child	Numeric		- Patient<br>- Hospital admission<br>- Diagnoses<br>- Procedures & surgeries<br>- Prescription
PROVIDER_ID	Unique reference number as present in the provider's linked data	String		- Patient<br>- Hospital admission<br>- Diagnoses<br>- Procedures & surgeries<br>- Prescription
L_ADM_REC	Unique admission record number<br><br>Used to link the child's admission records to the child's diagnoses and procedures during that admission<br>Generated by syntax script			- Hospital admission<br>- Diagnoses<br>- Procedures & surgeries
L_CASECON	Case or control status (apply status AFTER linkage is complete)<br><br>Exclusions:<br>- child with gestational age <23 weeks<br><br>Control with ICD10 Q code or ICD9 (-CM) 740-759 codes recorded. This is a child with a congenital anomaly code (ICD10 Q code, or ICD9-CM 740-759) in hospital	Numeric	1= Case<br>2= Control with no ICD10 Q code or ICD9(-CM) 740-759 codes recorded<br>3=Control with ICD10 Q code or ICD9(-CM) 740-759 codes recorded	- Patient<br>- Hospital admission<br>- Diagnoses<br>- Procedures & surgeries<br>- Prescription

Variable name	*Variable definition/ description*	*Variable format*	*Variable values*	*Tables[1]*
	discharge records that is not recorded as a EUROCAT case.<br><br>Children coded as 1 or 2 will be included in WP4 analysis.<br><br>Children coded as 3 are required for WP6			
L_SURG_DATE	Date of surgery / procedure<br><br>If date of surgery is missing, use the date of ICU admission as a proxy. If date of ICU is also missing, use date of discharge	DDMonYYYY		- Procedures & surgeries
L_SURG_YEAR	Year of surgery / procedure	YYYY		- Procedures & surgeries
L_SURG_AGE	Child's age at surgery <u>in days</u> (up to 10th birthday).<br><br>Subtract child's date of birth from date of surgery / procedure	Numeric	0= <1 day old<br>1= 1 day old<br>2= 2 days old etc<br>9999=surgery, but exact age not known	- Procedures & surgeries
L_SURG_CODE	Code for surgery / procedure performed during this hospital stay<br><br>As recorded in the hospital database	String		- Procedures & surgeries
SU_ANY	Any Surgery<br><br>As recorded in hospital database	Binary	0 = No<br>1 = Yes	- Procedures & surgeries
SU_FEEDING	Surgical Feeding Tube inserted<br>As recorded in hospital database	Binary	0 = No<br>1 = Yes	- Procedures & surgeries
SU_SB	Surgery for spina bifida<br>As recorded in hospital database	Binary	0 = No<br>1 = Yes	- Procedures & surgeries
SU_REVSHUNT	Revisions of shunt surgery – relevant to both spina bifida and hydrocephaly<br>As recorded in hospital database	Binary	0 = No<br>1 = Yes	- Procedures & surgeries
SU_EYE	Eye surgery<br>As recorded in hospital database	Binary	0 = No<br>1 = Yes	- Procedures & surgeries
SU_CARDIAC	Any Cardiac surgery<br>As recorded in hospital database	Binary	0 = No<br>1 = Yes	- Procedures & surgeries
SU_RASHKIND	Rashkind procedure<br>As recorded in hospital database	Binary	0 = No<br>1 = Yes	- Procedures & surgeries
SU_TGA_ARTERIAL	Arterial switch<br>As recorded in the hospital database	Binary	0 = No<br>1 = Yes	- Procedures & surgeries
SU_TGA_ATRIAL	Atrial switch<br>As recorded in the hospital database	Binary	0 = No<br>1 = Yes	- Procedures & surgeries
SU_VSD	Surgery for ventricular septal defect<br>As recorded in hospital database	Binary	0 = No<br>1 = Yes	- Procedures & surgeries
SU_ASD	Surgery for atrial septal defect<br>As recorded in hospital database	Binary	0 = No<br>1 = Yes	- Procedures & surgeries

Variable name	Variable definition/ description	Variable format	Variable values	Tables[1]
SU_AVSD	Surgery for atrioventricular septal defect<br>As recorded in hospital database	Binary	0 = No<br>1 = Yes	- Procedures & surgeries
SU_COARCT	Surgery for coarctation of aorta<br>As recorded in hospital database	Binary	0 = No<br>1 = Yes	- Procedures & surgeries
SU_PDA	Surgery for Patent ductus arteriosus (PDA) or catheter closure<br>As recorded in hospital database	Binary	0 = No<br>1 = Yes	- Procedures & surgeries
SU_BLALOCK	Blalock shunt<br>As recorded in hospital database	Binary	0 = No<br>1 = Yes	- Procedures & surgeries
SU_HEMIFON	Hemi-Fontan Surgery<br>As recorded in hospital database	Binary	0 = No<br>1 = Yes	- Procedures & surgeries
SU_FONTAN	Complete Fontan surgery<br>As recorded in hospital database	Binary	0 = No<br>1 = Yes	- Procedures & surgeries
SU_CLEFT	Cleft surgery<br>As recorded in hospital database	Binary	0 = No<br>1 = Yes	- Procedures & surgeries
SU_OESOPH	Surgery for oesophageal atresia<br>As recorded in hospital database	Binary	0 = No<br>1 = Yes	- Procedures & surgeries
SU_DILATION	Oesophageal atresia dilation<br>As recorded in hospital database	Binary	0 = No<br>1 = Yes	- Procedures & surgeries
SU_INTESTINE	Intestinal surgery<br>As recorded in hospital database	Binary	0 = No<br>1 = Yes	- Procedures & surgeries
SU_STOMY	Stoma Surgery<br>As recorded in the hospital database	Binary	0 = No<br>1 = Yes	- Procedures & surgeries
SU_KASAI	Liver/bile surgery (Kasai)<br>As recorded in hospital database	Binary	0 = No<br>1 = Yes	- Procedures & surgeries
SU_CDH	Surgery for diaphragmatic hernia<br>As recorded in hospital database	Binary	0 = No<br>1 = Yes	- Procedures & surgeries
SU_GASTRO	Surgery for gastroschisis<br>As recorded in hospital database	Binary	0 = No<br>1 = Yes	- Procedures & surgeries
SU_RENAL	Renal surgery<br>As recorded in hospital database	Binary	0 = No<br>1 = Yes	- Procedures & surgeries
SU_HYPOSPA	Surgery for hypospadias<br>As recorded in hospital database	Binary	0 = No<br>1 = Yes	- Procedures & surgeries
SU_TRANY	Transplantation (heart, kidney or liver)<br>As recorded in hospital database	Binary	0 = No<br>1 = Yes	- Procedures & surgeries
SU_TRHEART	Transplantation (heart)<br>As recorded in hospital database	Binary	0 = No<br>1 = Yes	- Procedures & surgeries
SU_TRKIDNEY	Transplantation (kidney)<br>As recorded in hospital database	Binary	0 = No<br>1 = Yes	- Procedures & surgeries
SU_TRLIVER	Transplantation (liver)	Binary	0 = No	- Procedures & surgeries

Variable name	***Variable definition/ description***	***Variable format***	***Variable values***	***Tables[1]***
	As recorded in hospital database		1 = Yes	
L_ICU_DATE_ADM	Date of admission to ICU	DDMonYYYY	. = Not recorded or not available for study	- Procedures & surgeries
L_ICU_DATE_DIS	Date moved from ICU to other ward	DDMonYYYY	. = Not recorded or not available for study	- Procedures & surgeries
L_ICU_YEAR	Year of admission to ICU	YYYY	. = Not recorded or not available for study	- Procedures & surgeries
L_ICU_AGE	Child's age at admission to ICU <u>in days</u> (up to 10th birthday).<br><br>Subtract child's date of birth from date of ICU admission	Numeric	0= <1 day old<br>1= 1 day old<br>2= 2 days old etc<br>9999= exact age not known<br>. = Not recorded by registry or not available for study	- Procedures & surgeries
**Table: Prescriptions**<br>***(for 5 conditions only: Asthma, Cardiac, Diabetes, Epilepsy and Infections)***<br>***<u>One row for each prescription.</u>***<br>***If multiple prescriptions, please provide prescription date and age of child at each prescription***				
L_CH_REC	Unique child record number<br><br>Generated by syntax script for all children (cases and controls), irrespective of whether the child has been in hospital<br><br>For children with a hospital admission, this is used to link admission, diagnoses, procedures & surgeries and prescriptions to child	Numeric		- Patient<br>- Hospital admission<br>- Diagnoses<br>- Procedures & surgeries<br>- Prescriptions
PROVIDER_ID	Unique reference number as present in the provider's linked data	String		- Patient<br>- Hospital admission<br>- Diagnoses<br>- Procedures & surgeries<br>- Prescription
L_CASECON	Case or control status (apply status AFTER linkage is complete)<br><br>Exclusions:<br>- child with gestational age <23 weeks<br><br>Control with ICD10 Q code or ICD9 (-CM) 740-759 codes recorded. This is a child with a congenital anomaly code (ICD10 Q code, or ICD9-CM 740-759) in hospital discharge records that is not recorded as a EUROCAT case.<br><br>Children coded as 1 or 2 will be included in WP4 analysis.<br><br>Children coded as 3 are required for WP6	Numeric	1= Case<br>2= Control with no ICD10 Q code or ICD9(-CM) 740-759 codes recorded<br>3=Control with ICD10 Q code or ICD9(-CM) 740-759 codes recorded	- Patient<br>- Hospital admission<br>- Diagnoses<br>- Procedures & surgeries<br>- Prescription
L_AGE_PX	Child's age at prescription <u>in complete days</u> (up to 10th birthday).<br><br>Subtract child's date of birth from the date prescription was issued/ dispensed	Numeric	0= <1 day old<br>1= 1 day old<br>2= 2 days old etc<br>9999= exact age not known<br>. = Not recorded or not available for study	- Prescriptions

Variable name	*Variable definition/ description*	*Variable format*	*Variable values*	*Tables[1]*
L_DATE_PX	Date prescription was issued/ dispensed	DDMonYYYY	. = Not recorded or not available for study	- Prescriptions
L_DRUG_CODE	Codes for medication: Anatomical Therapeutic Chemical classification (ATC), or Read codes	As recorded in local prescription database		- Prescriptions
DIA_ANY	Any prescribed antidiabetic medication<br><br>(ATC codes beginning A10)	Binary	0 = No<br>1 = Yes	- Prescriptions
DIA_INS	Any prescribed Insulin or insulin analogues<br><br>(ATC codes beginning A10A)	Binary	0 = No<br>1 = Yes	- Prescriptions
DIA_GLU_LOW	Any prescribed blood glucose lowering drugs, excluding insulins<br><br>(ATC codes beginning A10B)	Binary	0 = No<br>1 = Yes	- Prescriptions
INF_ANY	Any prescribed anti-infective medication<br><br>(ATC codes beginning J01-J05)	Binary	0 = No<br>1 = Yes	- Prescriptions
INF_ANTIBAC_SYS	Any prescribed antibacterials for systemic use<br><br>(ATC codes beginning J01)	Binary	0 = No<br>1 = Yes	- Prescriptions
INF_PEN	Any prescribed beta-lactam antibacterials, penicillins<br><br>(ATC codes beginning J01C)	Binary	0 = No<br>1 = Yes	- Prescriptions
INF_MAC	Any prescribed macrolides, lincosamides and streptogramins<br><br>(ATC codes beginning J01F)	Binary	0 = No<br>1 = Yes	- Prescriptions
INF_ANTIVIR_SYS	Any prescribed antivirals for systemic use<br><br>(ATC codes beginning J05)	Binary	0 = No<br>1 = Yes	- Prescriptions
EPI_ANY	Any prescribed antiepileptic drugs (AEDs)<br><br>(ATC codes beginning N03)	Binary	0 = No<br>1 = Yes	- Prescriptions
EPI_OLD	Any prescribed older AEDs<br><br>(ATC codes beginning N03AA phenobarbital, N03AB phenytoin, N03AE clonazepam, N03AF carbamazepine)	Binary	0 = No<br>1 = Yes	- Prescriptions
EPI_VPA	Any prescribed fatty acid derivatives<br><br>(ATC codes beginning N03AG, valproic acid)	Binary	0 = No<br>1 = Yes	- Prescriptions
EPI_NEW	Any prescribed newer AEDs<br><br>(ATC codes N03AX lamotrigine, topiramate, gabapentin, levetiracetam)	Binary	0 = No<br>1 = Yes	- Prescriptions
AST_ANY	Any prescribed antiasthmatic medications<br><br>(ATC codes beginning R03)	Binary	0 = No<br>1 = Yes	- Prescriptions

Variable name	Variable definition/ description	Variable format	Variable values	Tables[1]
AST_IN_B2	Any prescribed Inhaled β2- agonists<br><br>(ATC codes beginning R03AC)	Binary	0 = No<br>1 = Yes	- Prescriptions
AST_IN_CORT	Any prescribed Inhaled corticosteroids<br><br>(ATC codes beginning R03BA)	Binary	0 = No<br>1 = Yes	- Prescriptions
AST_ANTICHO	Any prescribed Anticholinergic inhaled medications<br><br>(ATC codes beginning R03BB)	Binary	0 = No<br>1 = Yes	- Prescriptions
AST_ANTIALL	Any prescribed Antiallergic agents, excl. corticosteroids<br><br>(ATC codes beginning R03BC)	Binary	0 = No<br>1 = Yes	- Prescriptions
AST_LEU	Any prescribed Leukotriene receptor antagonists<br><br>(ATC codes beginning R03DC)	Binary	0 = No<br>1 = Yes	- Prescriptions
CAR_ANY	Any prescribed cardiac medication, excluding quinidine and adrenalin pen<br><br>(ATC codes beginning C01-C03, C07-C09, excluding C01BA51, C01BA71, C01CA24)	Binary	0 = No<br>1 = Yes	- Prescriptions
CAR_VWC1	Any prescribed sodium channel blockers Vaughan Williams class (VWC) 1<br><br>(ATC codes beginning C01BA, C01BB and C01BC, excluding C01BA51, C01BA71 quinidine)	Binary	0 = No<br>1 = Yes	- Prescriptions
CAR_VWC2	Any prescribed betablockers VWC 2<br><br>(ATC codes beginning C07A)	Binary	0 = No<br>1 = Yes	- Prescriptions
CAR_VWC3	Any prescribed VWC 3 medications (amiodarone)<br><br>(ATC codes beginning C01BD)	Binary	0 = No<br>1 = Yes	- Prescriptions
CAR_VWC5	Any prescribed VWC 5 medications (digoxine)<br><br>(ATC codes beginning C01AA05)	Binary	0 = No<br>1 = Yes	- Prescriptions
CAR_HYP	Any prescribed medications for hypertension (calcium antagonists and agents acting on renin-angiotensin system)<br><br>(ATC codes beginning C08, C09)	Binary	0 = No<br>1 = Yes	- Prescriptions
CAR_DIU	Any prescribed diuretics<br><br>(ATC codes beginning C03)	Binary	0 = No<br>1 = Yes	- Prescriptions
L_GP_HOSP	Indicates if community pharmacy or hospital outpatient pharmacy prescription (for Italian registries only)	Numeric	1= Community pharmacy prescription<br>2= Hospital outpatient pharmacy prescription<br>3= Not available (for non-Italian registries)	- Prescriptions

Morris JK, *et al. BMJ Open* 2021; 11:e047859. doi: 10.1136/bmjopen-2020-047859
