# Supplementary material for: EUROlinkCAT protocol for a European population-based data linkage study investigating the survival, morbidity and education of children with congenital anomalies
Source: BMJ Open. 2021 Jun 28;11(6):e047859. doi: 10.1136/bmjopen-2020-047859 (PMC8240574; doi:10.1136/bmjopen-2020-047859)
Supplement: Author's manuscript [file bmjopen-2020-047859.draft_revisions.pdf]

BMJ Open is committed to open peer review. As part of this commitment we make the peer review history of every article we publish publicly available.

When an article is published we post the peer reviewers' comments and the authors' responses online. We also post the versions of the paper that were used during peer review. These are the versions that the peer review comments apply to.

The versions of the paper that follow are the versions that were submitted during the peer review process. They are not the versions of record or the final published versions. They should not be cited or distributed as the published version of this manuscript.

BMJ Open is an open access journal and the full, final, typeset and author-corrected version of record of the manuscript is available on our site with no access controls, subscription charges or pay-per-view fees (http://bmjopen.bmj.com).

If you have any questions on BMJ Open's open peer review process please email info.bmjopen@bmj.com

BMJ Open

EUROlinkCAT Protocol for a European population-based data linkage study investigating the survival, morbidity and education of children with congenital anomalies

Journal:	*BMJ Open*
Manuscript ID	bmjopen-2020-047859
Article Type:	Protocol
Date Submitted by the Author:	14-Dec-2020
Complete List of Authors:	Morris, Joan; St George's University of London, Population Health Research Institute<br>Garne, Ester; Hospital Lillebaelt Kolding, Region Syddanmark<br>Loane , Maria ; Ulster University Faculty of Life and Health Sciences<br>Barisic, Ingeborg; Klinika za dječje bolesti Zagreb<br>Densem, James; Biomedical Computing Limited<br>Latos-Bieleńska, Anna; Uniwersytet Medyczny imienia Karola Marcinkowskiego w Poznaniu Wydzial I Lekarski<br>Neville, Amanda; University Hospital of Ferrara, IMER Registry (Emila Romagna Registry of Birth Defects)<br>Pierini, Anna; Institute of Clinical Physiology National Research Council<br>Rankin, Judith; Newcastle University, Population Health Sciences Institute<br>Rissmann, Anke; Otto von Guericke University Medical Faculty, Malformation Monitoring Centre Saxony-Anhalt<br>de Walle, Hermien; University Medical Centre Groningen, Department of Genetics<br>Tan, Joachim; St George's University of London, Population Health Research Institute<br>Given, Joanne; Ulster University Faculty of Life and Health Sciences<br>Claridge, Hugh; St George's University of London, Population Health Research Institute
Keywords:	PAEDIATRICS, STATISTICS & RESEARCH METHODS, EPIDEMIOLOGY

SCHOLARONE™
Manuscripts

I, the Submitting Author has the right to grant and does grant on behalf of all authors of the Work (as defined in the below author licence), an exclusive licence and/or a non-exclusive licence for contributions from authors who are: i) UK Crown employees; ii) where BMJ has agreed a CC-BY licence shall apply, and/or iii) in accordance with the terms applicable for US Federal Government officers or employees acting as part of their official duties; on a worldwide, perpetual, irrevocable, royalty-free basis to BMJ Publishing Group Ltd ("BMJ") its licensees and where the relevant Journal is co-owned by BMJ to the co-owners of the Journal, to publish the Work in this journal and any other BMJ products and to exploit all rights, as set out in our licence.

The Submitting Author accepts and understands that any supply made under these terms is made by BMJ to the Submitting Author unless you are acting as an employee on behalf of your employer or a postgraduate student of an affiliated institution which is paying any applicable article publishing charge ("APC") for Open Access articles. Where the Submitting Author wishes to make the Work available on an Open Access basis (and intends to pay the relevant APC), the terms of reuse of such Open Access shall be governed by a Creative Commons licence – details of these licences and which Creative Commons licence will apply to this Work are set out in our licence referred to above.

Other than as permitted in any relevant BMJ Author's Self Archiving Policies, I confirm this Work has not been accepted for publication elsewhere, is not being considered for publication elsewhere and does not duplicate material already published. I confirm all authors consent to publication of this Work and authorise the granting of this licence.

For peer review only

EUROlinkCAT Protocol for a European population-based data linkage study investigating the survival, morbidity and education of children with congenital anomalies

Joan K Morris[1], Ester Garne[2], Maria Loane[3], Ingeborg Barišić[4], James Densem[5], Anna Latos-Bieleńska[6], Amanda Neville[7], Anna Pierini[8], Judith Rankin[9], Anke Rissmann[10], Hermien E.K. de Walle[11], Joachim Tan[1], Joanne Given[3], Hugh Claridge[1]

1. Population Health Research Institute, St George's, University of London, UK
2. Hospital Lillebaelt Kolding, Region Syddanmark, Denmark
3. Faculty of Life and Health Sciences, Ulster University, Northern Ireland, UK
4. Klinika za dječje bolesti Zagreb, Croatia
5. Biomedical Computing Limited, Battle, United Kingdom
6. Uniwersytet Medyczny im. Karola Marcinkowskiego w Poznaniu, Poznań, Poland
7. IMER Registry (Emila Romagna Registry of Birth Defects), University Hospital of Ferrara, Italy
8. National Research Council-Institute of Clinical Physiology (Consiglio Nazionale delle Ricerche-Istituto di Fisiologia Clinica), Pisa, Italy
9. Population Health Sciences Institute, Newcastle University, UK
10. Malformation Monitoring Centre Saxony-Anhalt, Medical Faculty Otto-von-Guericke-University Magdeburg, Magdeburg, Germany
11. University of Groningen, University Medical Center Groningen, Department of Genetics, Groningen, the Netherlands

Correspondence to Joan K Morris; jmorris@sgul.ac.uk

Word count: 5383

Keywords: Protocol; Congenital Anomalies; Linkage; Mortality; Morbidity

For peer review only

Abstract

Introduction

Congenital anomalies (CA) are a major cause of infant mortality, childhood morbidity and long-term disability. Over 130,000 children born in Europe every year will have a CA. This paper describes the EUROlinkCAT study, which is investigating the health and educational outcomes of children with CAs for the first 10 years of their lives.

Methods and analysis

EUROCAT is a European network of population-based registries for the epidemiological surveillance of CAs. EUROlinkCAT is using the EUROCAT infrastructure to support 22 EUROCAT registries in 14 countries to link their data on births with CAs to mortality, hospital discharge, prescription and educational databases. Once linked, each registry transforms their case data into a Common Data Model (CDM) format and they are then supplied with common STATA syntax scripts to analyse their data. The resulting aggregate tables and analysis results are submitted to a Central Results Repository (CRR) and meta-analyses are performed to summarise the results across all registries. The CRR currently contains data on 155,594 children with a CA followed up to age 10 from a population of 6 million births from 1995 to 2014.

Ethics

The CA registries have the required ethics permissions for routine surveillance and transmission of anonymised data to the EUROCAT central database. Each registry is responsible for applying for and obtaining additional ethics and other permissions required for their participation in EUROlinkCAT.

Dissemination

The CDM and associated documentation, including linkage and standardisation procedures, will be available post-EUROlinkCAT thus facilitating future local, national and EU-level analyses to improve health care. Recommendations to improve the accuracy of routinely collected data will be made. Findings will provide evidence to inform parents, health professionals, public health authorities and national treatment guidelines to optimise diagnosis, prevention and treatment for these children with a view to reducing health inequalities in Europe.

Article summary

Strengths and limitations of this study

- The implementation of a common data model enables the same centrally developed syntax script to be run in all registries which is efficient and ensures standardisation of analysis.

For peer review only

- The use of a reference population allows country differences to be adjusted for and enables more accurate comparisons of the burden of disease attributable to congenital anomalies across countries to be made.
- Merging births with congenital anomalies to their records in routine health care data enables a detailed evaluation of the accuracy of the data and enables improvements to be suggested.
- Being unable to share individual case data or aggregate data that might be disclosive reduces the power of the analysis that can be performed, particularly for rare anomalies.
- Only specific areas in Europe are represented, with a lack of data in particular from Eastern Europe.

For peer review only

Introduction

Over 130,000 children born in Europe every year will have major a CA, equivalent to 2.5% of all European births. CAs include structural defects, chromosomal anomalies and genetic syndromes. CAs are a leading cause of perinatal and infant mortality, especially in developed countries [1]. From 2003 to 2012, CAs were associated with about 40% of all infant deaths in Sweden and England [2]. There is a large variation in child death rates across Europe; in 2013 the child death rates (age 0-14 years) were 60% higher in the UK and Belgium compared to Sweden, with an additional 10 countries being 30% higher than Sweden [3]. To identify potentially preventable and remedial causes it is important to investigate the health inequalities in survival in children with CA across Europe.

It has been shown that relying on death certificates as a source of information on mortality due to CAs does not provide an accurate assessment of the survival for children with specific CAs. Death certificates state the direct or primary cause of death which may be infection, seizures or others and therefore may not mention the CA[4]. Copeland et al. (2007)[4] concluded that the only way to accurately study mortality and survival in children with rare CAs is to pool data across CA registries and link these to death registries.

Advances in fetal, neonatal and paediatric care have improved outcomes for individuals with some CAs, for example Down syndrome[5-7] and cardiac anomalies[8]. Several studies have shown that children with CAs account for a very high proportion of all hospital admissions [9, 10]. However, there is a lack of information on the length of hospital stays for children with specific CAs, with most studies concerning children with Down syndrome, orofacial clefts or congenital heart defects (CHD) [11]. Often hospital stays are investigated for the first two or three years of a child's life[12-15]. However, Wehby et al. (2012) showed that hospital admissions for those born with orofacial clefts were increased at all ages up to 60 years of age[16]. Rarely has length of hospital stay been related to other factors, such as social class. Two studies (Derrington 2013 in the USA to 3 years of age and Hung 2011 in Taiwan for all ages) both identified other factors such as ethnicity and socio-economic factors as important influences on the length of inpatient stays in children and adults with Down syndrome[15, 17].

The proportion of children born with a CA surviving beyond infancy is increasing [5, 6]. How these children are performing in school and their additional educational needs is therefore becoming increasingly important as there may be a growing population of children and young people requiring additional support and resources in the future. However, apart from the more common genetic syndromes, there is a paucity of information about this[18, 19]. The American Heart Association reviewed the literature on children with CHD and concluded that they are at an increased risk of developmental delay, even once the frequent occurrence of genetic syndromes has been taken into account, particularly for neonates or infants requiring open heart surgery[20]. Wehby et al. (2015) also showed that children with isolated orofacial clefts were at a much greater risk of low achievement at school than their classmates[21]. A systematic review of neurocognitive outcomes following general anaesthesia and surgery in children concluded that exposure to general anaesthesia in young children did affect their development in some neurocognitive domains [22]. However, the authors recommended that the effects of surgery should be considered separately for each specific anomaly.

EUROCAT (https://eu-rd-platform.jrc.ec.europa.eu/eurocat) is a European network of population-based CA registries, which started in 1979 and increased to include 39 registries in 21 countries covering more than 29% of European births (1.7 million) per year [23-25]. The main objectives of EUROCAT are to provide essential epidemiologic information and surveillance on CAs in Europe, to evaluate the effectiveness of primary prevention and to assess the impact of developments in prenatal screening[26, 27]. Hence the emphasis is on information collected up to a baby's first year of life.

The aim of EUROlinkCAT is to investigate the survival, morbidity and educational outcomes of children with specific CAs for the first 10 years of their lives by linking births with CAs in EUROCAT registries to electronic health care and education databases. The availability of population-based data on births with CAs across the EUROCAT network will enable survival, morbidity and education to be investigated for specific CAs as well as differences in these outcomes across Europe according to specific risk factors and social inequalities to be explored.

Electronic health care data are increasingly being used by researchers to investigate the epidemiology of CAs, rather than using information from CA registries. Such health care data have often been found to be incomplete[28-34]. A small number of registries will analyse the maternal pregnancy records for women registered as having had a termination of pregnancy for a fetal anomaly (TOPFA) in EUROCAT. This will enable the accuracy of routine information on TOPFAs to be evaluated. The accuracy of CA coding in live births will be evaluated by comparing the EUROCAT data for live births with the CA diagnosis from the electronic health care databases covering both in-patient and out-patient visits. The information on death certificates will be compared to the anomalies recorded in EUROCAT. Recommendations will be developed to enable the maximum

For peer review only

information from electronic health care data to be extracted for research purposes and to quantify the amount of data that cannot be obtained.

This paper describes the design of the study, the methods used to obtain and analyse the linked data and evaluates the first three years' progress of EUROlinkCAT.

Methods and analysis

Design and Setting

In 2017, all EUROCAT registries were invited to participate in the EUROlinkCAT study. Twenty-two registries from 14 countries agreed to participate and to link all live births with a CA registered in their registries and born from 1st January 1995-31st December 2014. Almost all EUROCAT registries send anonymised data on CAs occurring in all livebirths, fetal deaths from 20 weeks gestation and TOPFAs to the EUROCAT central database. Comprehensive coding instructions [35] and the use of the EUROCAT Data Management Program (EDMP) to import data into the central database ensure that standard variables, definitions and coding are used by all registries in the network. CAs are coded locally using the WHO International Statistical Classification of Diseases and Related Health Problems (ICD) 9th or 10th Revision with the British Paediatric Association (BPA) code extension offering more specificity (Table 1). Cases with minor anomalies only are excluded (see EUROCAT Guide 1.4, Minor Anomalies for Exclusion (version 14.10.14)). Registries can code up to nine anomalies for each case and provide additional information in the specified text fields. Based on the ICD-9 or ICD-10 codes present, cases are automatically assigned by EDMP to defined major CA subgroups in accordance with the EUROCAT guide 1.4. A case with more than one major anomaly may be assigned to more than one subgroup. Since 2015, the central database has been hosted by the European Commission Joint Research Centre in Ispra (Italy).

Tables 1 and 2 provide a list of the 81 EUROlinkCAT CA subgroups which include structural anomalies, genetic syndromes and chromosomal anomalies that will be investigated. There are 60 EUROCAT subgroups (Table 1) and an additional 21 new CA subgroups not defined in EUROCAT (Table 2). The subgroups have been identified as being reasonably homogeneous to provide meaningful information and also to be prevalent enough to enable sufficiently precise estimates to be obtained from the analyses. For example, the EUROCAT subgroup "Chromosomal" was not included as it includes all genetic syndromes, but specific syndromes such as Down syndrome (a EUROCAT subgroup) and Di George syndrome (a new subgroup) were included. For some analysis, such as mortality, Down syndrome children will be analysed according to whether they have a cardiac anomaly and/or a gastrointestinal anomaly, as these are common and are likely to influence their survival. As outcomes are expected to be more severe for children with multiple and more complex CAs, analyses are also performed separately for children with isolated anomalies or with multiple anomalies defined according to the methodology by Garne et al.[36] . Isolated anomalies are defined as a CA in one organ system only or with a known sequence where multiple CAs cascade

For peer review only

as a consequence of a single primary anomaly. Multiple anomalies are defined as two or more major structural CAs in different organ systems, where the pattern of anomalies has not been recognised as part of a syndrome or sequence.

For peer review only

Table 1: EUROCAT congenital anomaly subgroups in EUROlinkCAT

**EUROCAT Subgroups**	**ICD-10-BPA**	**ICD-9-BPA†**
**All anomalies ***	Q-chapter, D215, D821, D1810^, P350, P351, P371	74, 75, 27910, 2281^, 76076, 76280, 7710, 7711, 77121
**Structural anomalies**		
Spina Bifida	Q05	741
Hydrocephalus	Q03	7423
Severe microcephaly	Q02	7421
Congenital cataract	Q120	74332
Congenital Heart Defects (CHD)	Q20-Q26	745, 746, 7470-7474
Severe CHD	Q200, Q201, Q203, Q204, Q212, Q213, Q220, Q224, Q225, Q226, Q230, Q232, Q233, Q234, Q251, Q252, Q262	74500, 74510, 7452, 7453, 7456, 7461, 7462, 74600, 7463, 7465, 7466, 7467, 7471, 74720, 74742
Transposition of great vessels	Q203	74510
Ventricular Septal Defect	Q210	7454
ASD	Q211	7455
AVSD	Q212	7456
Tetralogy of Fallot	Q213	7452
Pulmonary valve stenosis	Q221	74601
Aortic valve atresia/stenosis	Q230	7463
Mitral valve anomalies	Q232, Q233	7465, 7466
Hypoplastic left heart	Q234	7467
Coarctation of aorta	Q251	7471
Patent ductus arteriosus (PDA) as only CHD in term infants (GA +37 weeks)	Q250	7470
Cystic adenomatous malformation of lung	Q3380	No code
Cleft lip with or without cleft palate	Q36, Q37	7491, 7492
Cleft palate	Q35	7490
Oesophageal atresia with/ without trachea-oesophageal fistula	Q390-Q391	75030-75031
Duodenal atresia or stenosis	Q410	75110
Atresia or stenosis of other parts of small intestine	Q411-Q418	75111-75112
Ano-rectal atresia and stenosis	Q420-Q423	75121-75124
Diaphragmatic hernia	Q790	75661
Gastroschisis	Q793	75671
Omphalocele	Q792	75670
Multicystic renal dysplasia	Q6140, Q6141	75316

For peer review only

Congenital hydronephrosis	Q620	75320
Hypospadias	Q54	75260
Limb reduction defects	Q71-Q73	7552-7554
Craniosynostosis	Q750	75600
**Chromosomal anomalies**		
Down syndrome	Q90	7580
Trisomy 13	Q914-Q917	7581
Trisomy 18	Q910-Q913	7582
Turner syndrome	Q96	75860, 75861, 75862, 75869
Klinefelter syndrome	Q980-Q984	7587
**Rare structural anomalies with a EUROCAT subgroup**		
Encephalocele	Q01	7420
Arhinencephaly / holoprosencephaly	Q041, Q042	74226
Anophthalmos / microphthalmos	Q110, Q111, Q112	7430, 7431
Anophthalmos	Q110, Q111	7430
Congenital glaucoma	Q150	74320
Anotia	Q160	74401
Common arterial truncus	Q200	74500
Double outlet right ventricle	Q201	No code
Single ventricle	Q204	7453
Triscuspid atresia and stenosis	Q224	7461
Ebstein's anomaly	Q225	7462
Pulmonary valve atresia	Q220	74600
Hypoplastic right heart	Q226	No code
Aortic atresia / interrupted aortic arch	Q252	74720
Total anomalous pulmonary venous return	Q262	74742
Choanal atresia	Q300	7480
Hirschsprung's disease	Q431	75130-75133
Atresia of bile ducts	Q442	75165
Annular pancreas	Q451	75172
Indeterminate sex	Q56	7527
Situs inversus	Q893	7593
VATER/VACTERL	Q8726	759895

For peer review only

Table 2: New congenital anomaly subgroups in EUROlinkCAT

**New subgroups for EUROlinkCAT**	**ICD-10-BPA**	**ICD-9-BPA†**
Structural anomalies		
Anomalies of corpus callosum	Q040	74221
Anomalies of intestinal fixation	Q433	7514
Unilateral renal agenesis	Q600	No code
Accessory kidney	Q630	75330
Bladder exstrophy	Q641	7535
Epispadia	Q640	75261
Posterior urethral valves	Q6420	75360
Prune Belly	Q794	75672
Arthrogryposis multiplex congenita	Q743	75580
Genetic syndromes		
Di George syndrome	D821	27910
Goldenhar syndrome	Q8704	75606
Cornelia de Lange syndrome	Q8712	759821
Noonan syndrome	Q8714	759896
Prader-Willi	Q8715	759872
Beckwith-Wiedemann syndrome	Q8730	759874
Williams syndrome	Q8784	No code
Angelman syndrome	Q8785	No code
Chromosomal anomalies		
Wolff-Hirschhorn syndrome	Q933	75832
Cri-du chat syndrome	Q934	75831
Karyotype XXX	Q970	75885
Sequences		
Pierre-Robin sequence	Q8708	75603

Linkage

Table 3 provides details of the linkages originally planned by the EUROCAT registries and the current linkages occurring (as of August 2020). The reasons why some registries could not link their data are explored in detail in another paper submitted for publication - they include not being able to obtain the necessary permissions, relevant outcomes not being recorded in specific data sources and the time scale for the data supply being after the end of the study's funding. Currently, 19 registries are linking their data to mortality records, 15 plan to link to hospital in-patient records and seven to prescription records for the work package that will consider morbidity for children born with a CA. At

For peer review only

the time of writing, nine registries plan to link their information on children with CAs to education records. To evaluate the accuracy of the routine health care data, five registries are additionally linking to out-patient data and four will also link to pregnancy information recorded in the mother's health records about TOPFAs. The 19 registries survey over 6 million births in the population.

Table 3: EUROCAT Congenital anomaly registries in EUROlinkCAT: start year, births in the population up to 2014, live births with an anomaly in the study period and ability to link to mortality, health care, prescription and education data

For peer review only

Congenital Anomaly Registry	Planned start year	Actual start year	Number of live births with an anomaly		Linkages Occurring (Y), Not occurring (N) and Planned but no longer occurring (¥)						
			Planned to be linked	Linked to mortality data by 8/2020	Mortality	Hospital Data for Child		Hospital Data for Mother		Prescriptions	Education
						In-patient	Out-patient	In-patient	Out-patient		
Belgium: Antwerp	1995	1997	8083	7865	Y	N	N	N	N	N	N
Croatia: Zagreb	1995	2011	2232	441†	¥	Y	¥	N	N		N
Denmark: Funen	1995	1995	2418	2425	Y	Y	Y	Y	Y	Y	Y
Finland	1995	1995	44869	42861	Y	Y	Y	Y	Y	Y	Y
France: Île de la Réunion	2002	NC	3855	NC	¥	¥	N	N	N	N	N
France: Paris	1997	1995	13335	11623	Y	N	N	N	N	N	N
Germany: Saxony-Anhalt	1995	2005	8821	8698	Y	N	N	N	N	N	N
Italy: Emilia Romagna	1995	2008	11447	7327	Y	Y	N	N	N	Y	Y
Italy: Tuscany	1995	2005	9827	5187	Y	Y	Y	N	N	Y	¥
Malta	1995	2005	2470	2718	Y	N	N	N	N	N	N
Netherlands: Northern	1995	2005	8567	8325	Y	Y	Y	Y	Y	Y	N
Norway	1999	1999	26938	27201	Y	N	N	N	N	N	N
Portugal: South	1995	2000*	3425*	2447*	N	Y	N	N	N	N	N
Spain: Basque Country	1995	1995	4883	5904	Y	¥	N	N	N	¥	N
Spain: Valencian Region	2007	2007	7438	7389	Y	Y	N	N	N	Y	N
UK: East Midlands and South Yorkshire	1998	AL	18549	AL	Y	Y	N	N	N	¥	Y
UK: Northern England	2000	AL	8617	AL	Y	Y	N	N	N	¥	Y
UK: South West England	2005	AL	11671	AL	Y	Y	N	N	N	¥	Y
UK: Thames Valley	1995	AL	5142	AL	Y	Y	N	N	N	¥	Y
UK: Wales	1998	1998	18239	18128	Y	Y	Y	Y	Y	Y	Y
UK: Wessex	1995	AL	7771	AL	Y	Y	N	N	N	¥	Y
Ukraine: West	2005	2006	6166	5835	Y	Y	N	N	N	N	N
Total			234763	155594†	19	15	5	4	4	7	9

¥: Linkages are no longer planned as of August 2020

†: The 441 cases in Zagreb were not included in analyses due to poor quality of the mortality linkage

The registry in Basque was unable to complete the planned linkages to hospital data due to COVID-19

NC: Linkages could not be completed

AL: Awaiting linkage as of August 2020

*: Values for WP4 morbidity linkage have been provided, as mortality linkage was never planned

For peer review only

For peer review only - http://bmjopen.bmj.com/site/about/guidelines.xhtml

For the evaluation of survival and morbidity, each child will be followed-up for a maximum of ten years. This age cut-off has been chosen to enable enough children to be identified and followed-up; a longer follow-up would mean fewer children would be eligible as currently national or local electronic health care record sources often do not go back more than 10 years. For education the maximum follow-up is until the end of compulsory school age (typically 16 across participating countries), although for some registers data are only available for a shorter period of follow-up. The longer follow-up was chosen in Finland where there are no national education tests and national data on education attainment are available at the age of 15-16 (9th grade).

Reference Population

Where possible, each registry obtained information from electronic health care records, prescription records and education records on children without a CA. The definition of these “control” cohorts will vary according to the registry, ranging from all children in the same population covered by the registry to a 10% random sample of children stratified by birth year and child’s sex. The use of such a reference population is essential in interpreting differences across countries, as it will provide information on key outcomes, such as duration of hospital stays and medication prescribing, on children without reported anomalies, which is expected to vary by country. Table 4 provides information on the reference populations being identified.

Table 4: Use of a reference population in morbidity and education analyses

Congenital Anomaly Registry	Reference Population
Croatia: Zagreb	Sample of children
Denmark: Funen	Whole Population
Finland	Whole Population
France: Île de la Réunion	Not provided
Italy: Emilia Romagna	Whole Population
Italy: Tuscany	10% of population
Netherlands: Northern	10% of Population
Portugal: South	Sample of children
Spain: Basque	Not provided
Spain: Valencian Region	Whole Population
UK: East Midlands and South Yorkshire	Aggregate data from population for morbidity and population sample for Education
UK: Northern England	Aggregate data from population for morbidity and population sample for Education
UK: South West England	Aggregate data from population for morbidity and population sample for Education
UK: Thames Valley	Aggregate data from population for morbidity and population sample for Education
UK: Wales	Whole Population
UK: Wessex	Aggregate data from population for morbidity and population sample for Education

For peer review only

Ukraine: West	No longer in morbidity study

Standardisation and Common Data Models

EUROCAT registries submit 96 core and non-core variables to the EUROCAT central database providing pseudonymised information on the baby and mother, diagnosis, karyotype (if known), exposure, family history, and socio-demographic details. These have already been standardised and Table 5 lists the 52 variables of which 34 are core variables and their common coding scheme that are used in the EUROlinkCAT study. In contrast, all the data obtained from linkage have to be standardised to a common format, as the health care and educational systems across Europe use different native languages and coding classification schemes. To do this, each registry provided their data dictionaries describing the variables in their local databases including the variable names, format, definitions, and coding schemes.

Table 5: Standardised variables from the EUROCAT database

	**EDMP Variables used (core variables are shaded in blue)**	
**Baby and Mother**		
1	CENTRE	Centre Number
2	NUMLOC	Local ID of case
3	BIRTH_DATE	Date of Birth
4	SEX	Sex
5	NBRBABY	Number of babies delivered
6	SP_TWIN	Specify twin type of birth, like or unlike, zygosity
7	NBRMALF	Number of malformed in multiple set
8	TYPE	Type of birth
9	CIVREG	Civil registration status
10	WEIGHT	Birth weight
11	GESTLENGTH	Length of gestation in completed weeks
12	SURVIVAL	Survival beyond one week of age
13	DEATH_DATE	Date of death
14	DATEMO	Date of birth of mother
15	AGEMO	Age of mother at delivery
16*	BMI	Maternal Body Mass Index
17	RESIDMO	Mother's residence code
**Diagnosis**		
19**	WHENDISC	When discovered
20	CONDISC	Condition at discovery
21	AGEDISC	If prenatally diagnosed, gestational age at discovery
22	FIRST PRE	First positive prenatal test
24	KARYO	Karyotype of infant/fetus
25	SP_KARYO	Specify karyotype
26*	GENTEST	Genetic Test
27*	SP_GENTEST	Specify genetic test
28	PM	Post mortem examination
29**	SURGERY	First surgery for malformation performed or planned
30	SYNDROME	Syndrome
31	SP_SYNDROME	Specify Syndrome
32	MALFO1	malformation
33	SP_MALFO1	Specify malformation
34	MALFO2	As MALFO1
35	SP_MALFO2	Specify malformation
36	MALFO3	As MALFO1
37	SP_MALFO3	Specify malformation

For peer review only - http://bmjopen.bmj.com/site/about/guidelines.xhtml

38	MALFO4	As MALFO1
39	SP_MALFO4	Specify malformation
40	MALFO5	As MALFO1
41	SP_MALFO5	Specify malformation
42	MALFO6	As MALFO1
43	SP_MALFO6	Specify malformation
44	MALFO7	As MALFO1
45	SP_MALFO7	Specify malformation
46	MALFO8	As MALFO1
47	SP_MALFO8	Specify malformation
57#	OMIM	OMIM code / Type of Mendelian Inheritance
**Exposure and family history**		
58**	ASSCONCEPT	Assisted conception (where available)
59##	OCCUPMO	Mother's occupation at time of conception
**Sociodemographic**		
91	MATEDU	Maternal education
92	SOCM	Socioeconomic status of mother
93	SOCF	Socioeconomic status of father
94	MIGRANT	Migrant status
**Derived variables**		
	Byear	Year of birth
	birth_type	Livebirth, Stillbirth, Spontaneous abortion, TOPFA, Not known Definitions of stillbirths and spontaneous abortions vary between regions. This variable recodes birth type according to EUROCAT's specifications: cases with gestational age ≥ 20 weeks are re-coded as "stillbirths" (irrespective of the local definition of stillbirth/spontaneous abortion).
	casestatus	Only cases with casestatus = 1 or 2
	al1-al114	EUROCAT subgroups: (0 = No, 1 = Yes). Based on EUROCAT coding in Guide 1.4
	mult_malf	Algorithm for case classification into isolated and multiple anomalies

Table 6 shows how the variable sex (male and female) is coded for children in the different registries with different variable names, different formats and different coding schemes. For each sub-study in EUROlinkCAT, a common data model (CDM) containing all variables required for its analyses was developed. All the EUROlinkCAT CDMs contain the variable L_CH_SEX, defined as "sex of child" and with a coding scheme in integer format of 1=male, 2=female, 3=Indeterminate, 9=Not known '.'= Not recorded or not available for study. Ulster University (UU) used the information in each registry's data dictionary to create the new EUROlinkCAT "standardised" L_CH_SEX variable. UU, in collaboration with the registries, created registry-specific syntax scripts to standardise all the variables in the EUROlinkCAT CDMs. Appendices 1, 2 and 3 list all the variables included in the sub-studies (mortality, electronic health care records and prescription records).

Table 6: Coding of male or female of the live births in different linked databases in EUROlinkCAT

		Code	
**Centre**	**Variable name**	**Male**	**Female**
UK: Wales	DEC_SEX_CD	1	2
Germany: Saxony-Anhalt	EF306	1	2

For peer review only

Finland	SUKUP	1	2
Italy: Tuscany	SESSO	1	2
France: Île de la Réunion	SexeDefunt	1	2
France: Paris	SexeDefunt	1	2
Netherlands: Northern	geslacht	1	2
Croatia: Zagreb	GENDER	M or 1	F or 2
Ukraine: West	CH_SEX	1	2
Belgium: Antwerp	SEX	1	2
Norway	KJONN	1	2
UK: England	sex	1	2
Spain: Basque Country	SEXO	1	6
Spain: Valencian Region	SEXO	1	6
Malta	gender	M	F
Italy: Emilia Romagna	SEX	M	F
Denmark: Funen	C_SEX	M	K

The CDMs also specify how the data are stored. For mortality, all the relevant variables occur only once for each EUROCAT case and are stored in the same data file (or table) (Figure 1). However, when analysing hospital admissions, each child may have more than one admission and for each admission may receive more than one diagnosis. Therefore, the hospital admissions data are stored in a separate data file (or table) from the diagnoses data and separately from the EUROCAT data on the child; each data file contains a reference key which serves to link all records belonging to one person for analysis (see Figure 2). The standardisation syntax scripts from UU specify the separate data files (or tables) for each linkage containing all the variables in the CDMs.

UU included validation routines in the syntax scripts to determine if the data have been correctly transformed. For example, it is checked that a date of death does not occur prior to the date of birth; whilst its primary purpose is to ensure data had been standardised correctly, it can also reveal any errors in the linked data.

A CDM is not being defined for the linked education data as there is limited scope for comparison and pooling of data across countries. This is due in part to inherent differences in the educational stages and systems, the variability in data available, and fewer registries being able to participate (5 of the registries are from England, and 1 from Wales, Denmark, Italy and Finland).

Work of the Standardisation Committee

In addition to defining the CDM and its structure, the EUROlinkCAT Standardisation Committee was responsible for taking other decisions, usually in consideration of local data characteristics, to ensure

For peer review only - http://bmjopen.bmj.com/site/about/guidelines.xhtml

that data were meaningful and comparable across registries. The most important issues are listed below:

i. **Inclusion according to gestational age (GA) at birth**: For the mortality study all livebirths with a GA below 24 weeks were excluded, as these cases could have been miscoded terminations of pregnancy with signs of life at birth. However, after running the mortality analysis, it was noticed locally that there were survivors in EUROCAT registries at GA 23 weeks. For the morbidity studies, the exclusion criterion was lowered to be below 23 weeks.

ii. **Strength of linkage**: The success of data linkage depended on the matching method and type of personal identifiers used. Where a national unique identifier (ID) was available (e.g. Denmark, Finland) over 99% of cases were matched, but success rates were generally lower when intermediary databases and a combination of other identifiers (e.g. names, postcodes) were required to establish a match, particularly if these were incomplete or incorrect. A standard way of evaluating confidence in a match had to be developed so that decisions on inclusion for analyses could be consistently made, in order to avoid bias.

iii. **Gestational age groups**: The gestational age at birth was categorised into <28 weeks, 28-31 weeks, 32-36 weeks and 37+ weeks when analysing survival, but due to small numbers of survivors at under 28 weeks gestation, the two lowest gestational age categories were combined to <32 weeks.

iv. **Birth weight**: Birth weight was categorised into very low birth weight '<1500 g', low birth weight '≥1500 g to <2500 g', normal birth weight '≥2500 g to 3999 g' and high birth weight `≥4000 g'. Births <1000 g were not distinguished from those between 1000-1499 g as there were too few cases for the data to be analysed accurately.

v. **Singletons vs. multiples**: There is uncertainty about whether the survival in twins with CAs is lower than that in singletons [37-40]. Hence, all survival analyses were performed on singletons alone and then multiples and singletons combined (multiples were not analysed alone as for many registries small numbers would limit the analyses that could be performed). This enables the survival of singletons and multiples in children with CAs to be analysed in detail. When examining morbidity, multiplicity was treated as one of the risk factors for increased risk of hospitalisations and lengths of stay to enable any association to be analysed, but with less detail than for survival. The majority of analyses included singletons and multiples combined.

vi. **Prenatal diagnoses**: The gestational age at prenatal diagnoses was categorised into <22 weeks, 22-31 weeks, 32+ weeks, gestational age not known and no prenatal diagnosis. For Finland, the gestational age was often not recorded, only the trimester of diagnoses. Finland's first trimester diagnoses (week 0 to week 12) mapped exactly to the EUROlinkCAT <22 weeks category. It was decided that Finland's 2nd trimester (week 13 to week 27) diagnoses were assumed to occur at 22-31 weeks and 3rd trimester (week 28 onwards) diagnoses occurred at 32+ weeks. These assumptions were also checked based

For peer review only

on the distribution of those cases in Finland with a “known” age at discovery and the assumptions held.

vii. **Length of stay**: The length of stay (LOS) of the child in hospital was calculated after excluding the stay associated with the birth. Methods of identifying the birth stay varied in different countries. For hospital admissions where admission and discharge occurred on the same day, the LOS was considered to be 0.5 days. If an admission record was missing a discharge date, then discharge date = date of admission + 2*(date of latest procedure – date of admission). The date of discharge was set to the date of the child’s 10th birthday or the end of the study period if it was after either of these two dates.

viii. **Socio-economic status (SES)**: All registries had different variables that could be considered to be a measure of the mother’s SES. The variables included maternal occupation, maternal education and index of multiple deprivation derived from residential codes at birth. Registries were asked to select the variable they believed was the most relevant and to re-code their selected SES proxy variable into three groups of approximately equal proportion to enable comparing between, for example, mothers in the highest group to mothers in the lowest group. The effect of SES on survival would be analysed using Cox proportional hazard models within each registry. However, only 7 registries were able to provide a proxy SES variable that was reasonably complete for some or all of the time period of the study. It was also planned to investigate the association between risk factors such as birth weight after adjusting for SES, but due to the lack of information on SES this was not included in further multivariable analyses.

ix. **Maternal country of birth**: It was determined that the maternal country of birth variable would be used as a proxy for non-European ethnic origin, as we were aware that ethnic origin is poorly recorded. However, for those registries with reasonably complete data on this, almost 100% of children were reported as being of European ethnic origin. Therefore, this variable was not included in subsequent analyses as the number of children considered as “Non-European ethnic origin” was too small to analyse.

x. **Cause of death:** cause of death based on the death certificates was classified for deaths <1 year and for 1-9 years separately. Death related to preterm birth is very common in the first year after birth, but not as relevant to children at 1-9 years of age. Injuries and poisoning are more common after the first year. The main causes of deaths were classified into 6 groups for deaths <1 year and 11 groups for children aged 1-9 years. When working with the results tables it was clear that many of these classification groups included many small numbers and data could not be extracted from the databases. For some registries it was only possible to give cause of death as either “congenital anomaly” or else “any other cause of death”.

xi. **Surgery**: A number of different coding systems were used across registries to code surgeries and other procedures (e.g. NCSP by NOMESCO, ICD-9-CM, OPCS-4). Frequency lists for all codes describing surgeries and other procedures were obtained from the linked datasets. Two paediatricians then independently determined if a code was a surgery or for another procedure and then a consensus between the two clinicians was

For peer review only

reached over codes classified as codes for surgeries. Further sub-division into anomaly-specific surgeries was carried out for anomalies for which specific surgeries could be identified that would be expected to be performed on these children.

xii. **Intensive care**: It was planned to analyse the number of days in intensive care, however only five registries could provide this. Therefore, only whether a child had ever been admitted to intensive care was analysed rather than their length of stay.

xiii. **Ventilation**: It was planned to analyse the number of days on ventilation. However, as it was decided that the length of stay in intensive care was not going to be analysed, the same decision was made for ventilation and only whether a child had ever been on ventilation was analysed.

Assessment of Quality of Linkage and Quality of Linked Data

Syntax scripts were developed centrally by St George's, University of London (SGUL) to evaluate the accuracy of the linkage and identify any factors leading to missed links (for example deaths within the first week of life). For each registry, the proportion of births in any single year of data that have not been linked is calculated and the data from any year with less than 85% of cases linked will be excluded from further analyses. Secondly, the quality of linked data items was also evaluated: a variable that was >20% missing in a year would be excluded from any analysis in which it featured; and variables that were recorded by both the CA registry and the linked database would be compared for agreement, by year. In general, it was found that data quality was poorer in the earlier years and tended to improve over time; however if data quality fluctuated across the years, then only the longest consecutive period where quality was above the threshold would be analysed.

Statistical Analysis

Protocols and syntax scripts are developed centrally to create aggregate data and perform specific analyses on the individual cases in each standardised data set in STATA (Version 13 and upwards). This allows each register to submit aggregated data and analytical results (e.g. Kaplan-Meier estimates, hazard ratios and confidence intervals), rather than individual case data, to the EUROlinkCAT CRR at UU, UK using a secure web platform. UU collates the aggregate data and results and provides these data to the researchers responsible for the different analyses and publications. Multi-centre European analyses will be performed by combining the individual registries' aggregated data and analytic results, using meta-analytic techniques. Additional work is required to develop suitable models for combining survival data from several registries when the sample sizes are very small as observed in many registries.

Small Number Restrictions (Statistical Disclosure Control)

Four countries have limitations on the release of aggregate data and analytic results if the numbers of births involved are very small (generally under eight births). This situation arises in many analyses

For peer review only

involving specific CAs, as CAs are rare, with some affecting less than 1 in 10,000 livebirths. Solutions to enable the maximum amount of data to be included in all multi-centre European analyses varied according to country. The Northern Netherlands released data if all exported results were rounded to the nearest 5. Rounding all frequencies ensures that original numbers cannot be inferred. For Denmark, a few named researchers at SGUL and UU were allowed access to the aggregate data for the purpose of collating and including in pooled-analysis, on condition that it was securely stored and processed; that any individual results involving fewer than five people were not released; and that personal identification was not possible from any released results. The SAIL databank (Wales) provided data to the CRR with the requirement that aggregate data on fewer than five people were not released and could not be calculated from any information in the public domain. The registry from Antwerp, Belgium could not release any information on three or fewer cases.

Patient and Public Involvement

A series of focus groups has been held in different European countries involving parents with a child with one of four pre-defined CAs with different health problems covering learning difficulties, physical disabilities, visible defects and non-visible defects with higher mortality. The four anomalies selected were: CHD requiring surgery (referred to as severe CHD – a usually non-visible defect with high mortality), cleft lip (CL; a visible defect often with speech problems), spina bifida (SB; a physical disability with associated incontinence problems) and Down syndrome (DS; Trisomy 21; a visible defect with learning difficulties and often associated with CHD). The focus groups have investigated parental experiences of having a child with one of the above anomalies and assessed parental research priorities ADD when paper published.

In addition, a European survey concerning the diagnosis, medical care, education and everyday life will be distributed to parents across Europe with children with the same four CAs as described above. Registries will ensure the questions in the survey are appropriate for their country (for example the provision of health services, given how this differs in various European countries) and will translate the survey into their native language with back translation to confirm the accuracy of the translation. The aim is for the survey to be distributed via social media by parent support groups across Europe to engage with a wide spectrum of parents.

Discussion

The aggregate data and results from the CRR in EUROlinkCAT will provide important information on the survival, morbidity and education of children born with a CA in Europe. Researchers in each CA registry will be encouraged to also perform specific local analysis, in order to fully exploit the research potential of linked datasets. The establishment of a method of standardising data from each registry linkage into a CDM provides valuable infrastructure enabling future multi-national studies to be performed in an efficient manner and new registries to become involved.

For peer review only

The strength of this study is that the researchers are a multidisciplinary group, many of whom have collaborated successfully for many years through being members of EUROCAT. In addition, EUROlinkCAT is able to build on all the standardisation procedures already established in EUROCAT. The implementation of a CDM enables the same centrally developed syntax script to be run in all the different registries which is efficient and also ensures standardisation of analysis across the registries. The use of a reference population when analysing health care data will aid in the identification of the source of differences between registries (for instance average LOS in hospitals will differ) and therefore enable us to better quantify the burden of disease attributable to CAs in each country. Comparisons of the accuracy of health care databases with respect to recording CA cases will be informative and enable improvements in those areas with less accurate data. We will also be developing recommendations on how to use the available health care data in an optimal way to provide information on children with CAs in areas without active CA registries.

One of the challenges of EUROlinkCAT is the ability of the CA registries to link their data to external data sources due to different local data information governance issues and the availability of suitable electronic health care databases. This requires flexibility in including registries in only specific sub-projects and acceptance that not all registries may be able to perform the linkages planned. In addition, some registries require support from other partners in all aspects of the project, including applying for ethics permissions, adapting protocols, standardising data and running statistical syntax scripts. The restriction of not being able to share individual case data and also aggregate data that might be disclosive or identifiable means that all analyses must be performed locally using a generic modelling strategy. This does limit the use of iterative procedures to explore data in detail. The major limitation to the study is that only specific areas in Europe are represented, with a lack of data in particular from Eastern Europe. Interpretation of differences across Europe is challenging as it will be essential to interpret results in the light of knowledge about the differences in health care and education practices across Europe.

The EUROlinkCAT project will enable important hypotheses concerning the survival, health and education of children with CAs in Europe to be investigated. The standardised methods and CDMs will all be available freely on the EUROlinkCAT website and will be available for use in future research projects to benefit from and build on this work, so as to enable other multi-centre European projects to exploit routine health care data available in Europe.

Ethics and dissemination

The CA registries have the required ethics permissions and procedures for routine surveillance, data collection and transmission of anonymised data to the EUROCAT central database, according to national guidelines. Local registries follow national legislation as to whether parental consent is needed for registration of babies with anomalies[41]. Each registry was responsible for applying for

For peer review only

and obtaining the additional ethics and other permissions (e.g. data sharing agreements) required to link and analyse their data for EUROlinkCAT. This was a lengthy process in some countries as the original data collection did not include expectation or consent for the data to be used in research, and a new legal basis had to be established. UU obtained ethics permission for the CRR. Additional assurances and procedures were adopted by registries (e.g. publication of privacy notices) to ensure compliance with the General Data Protection Regulation (GDPR) which came into force on 25 April 2018 in EU countries. A checklist of minimum specifications for data storage/backup was completed by each registry.

Each registry participating in the focus groups with parents was responsible for ensuring the correct ethics approvals were in place. Similarly, the registries participating in the dissemination of the parents' survey will be responsible for ensuring the necessary ethics permissions are obtained.

An Ethics and Data Protection Advisory Board (EDPB) consisting of three independent advisors with the relevant expertise monitor all ethical considerations in this project.

The CRR will be used for multiple studies and the results from these will be disseminated in peer reviewed papers and conference presentations. It is hoped that the experience gained with distributing the parents' survey using parent support groups and social media will also lead to development of a framework to enable dissemination of results to be made more directly to parents. In addition, a series of reports will be written including recommendations for improving the collection and analysis of data on CAs in routinely collected data in the health care databases.

References

1. Boyle, B., et al., *Estimating Global Burden of Disease due to congenital anomaly: an analysis of European data.* Arch Dis Child Fetal Neonatal Ed, 2018. **103**(1): p. F22-f28.
2. Zylbersztejn, A., et al., *Child mortality in England compared with Sweden: a birth cohort study.* Lancet, 2018. **391**(10134): p. 2008-2018.
3. Wang, H., et al., *Global, regional, and national levels of neonatal, infant, and under-5 mortality during 1990-2013: a systematic analysis for the Global Burden of Disease Study 2013.* Lancet, 2014. **384**(9947): p. 957-79.
4. Copeland, G.E. and R.S. Kirby, *Using birth defects registry data to evaluate infant and childhood mortality associated with birth defects: An alternative to traditional mortality assessment using underlying cause of death statistics.* Birth Defects Res A Clin Mol Teratol, 2007. **79**(11): p. 792-797.
5. Wu, J. and J.K. Morris, *The population prevalence of Down's syndrome in England and Wales in 2011.* Eur J Hum Genet, 2013. **21**(9): p. 1016-9.
6. Tennant, P.W., et al., *20-year survival of children born with congenital anomalies: a population-based study.* Lancet, 2010. **375**(9715): p. 649-56.
7. Brodwall, K., et al., *The five-year survival of children with Down syndrome in Norway 1994–2009 differed by associated congenital heart defects and extracardiac malformations.* Acta Paediatrica, 2018. **107**(5): p. 845-853.

For peer review only

8. Boneva, R.S., et al., *Mortality Associated With Congenital Heart Defects in the United States.* Circulation, 2001. **103**(19): p. 2376-2381.
9. Muranjan, M. and P. Vijayalakshmi, *The Unforeseen Toll of Birth Defects and their Economic Burden at a Tertiary Care Public Institute in Mumbai.* Ind J Pediatr, 2014. **81**(10): p. 1005-1009.
10. McCandless, S.E., J.W. Brunger, and S.B. Cassidy, *The burden of genetic disease on inpatient care in a children's hospital.* Am J Hum Genet, 2004. **74**(1): p. 121-7.
11. Shetty, S., et al., *Length of stay and cost analysis of neonates undergoing surgery at a tertiary neonatal unit in England.* Ann R Coll Surg Engl, 2016. **98**(1): p. 56-60.
12. Weiss, J., et al., *Hospital use and associated costs of children aged zero-to-two years with craniofacial malformations in Massachusetts.* Birth Defects Res A Clin Mol Teratol, 2009. **85**(11): p. 925-34.
13. Fitzsimons, K.J., et al., *Hospital care of children with a cleft in England.* Arch Dis Child, 2013. **98**(12): p. 970-974.
14. So, S.A., R.C. Urbano, and R.M. Hodapp, *Hospitalizations of infants and young children with Down syndrome: evidence from inpatient person-records from a statewide administrative database.* J Intellect Disabil Res, 2007. **51**(Pt 12): p. 1030-8.
15. Derrington, T.M., et al., *Racial/ethnic differences in hospital use and cost among a statewide population of children with Down syndrome.* Res Dev Disabil, 2013. **34**(10): p. 3276-87.
16. Wehby, G.L., et al., *The effects of oral clefts on hospital use throughout the lifespan.* BMC Health Services Research, 2012. **12**(1): p. 58.
17. Hung, W.J., et al., *Cost of hospitalization and length of stay in people with Down syndrome: evidence from a national hospital discharge claims database.* Res Dev Disabil, 2011. **32**(5): p. 1709-13.
18. O'Toole, C., et al., *Parent-mediated interventions for promoting communication and language development in young children with Down syndrome.* The Cochrane database of systematic reviews, 2018. **10**(10): p. CD012089-CD012089.
19. Iyer, N.P., et al., *Outcome of fetuses with Turner syndrome: a 10-year congenital anomaly register based study.* J Matern Fetal Neonatal Med, 2012. **25**(1): p. 68-73.
20. Marino, B.S., et al., *Neurodevelopmental outcomes in children with congenital heart disease: evaluation and management: a scientific statement from the American Heart Association.* Circulation, 2012. **126**(9): p. 1143-72.
21. Wehby, G.L., et al., *Children with oral clefts are at greater risk for persistent low achievement in school than classmates.* Arch Dis Child, 2015. **100**(12): p. 1148-54.
22. Clausen, N.G., S. Kähler, and T.G. Hansen, *Systematic review of the neurocognitive outcomes used in studies of paediatric anaesthesia neurotoxicity.* Br J Anaesth, 2018. **120**(6): p. 1255-1273.
23. Boyd, P.A., et al., *Paper 1: The EUROCAT network—organization and processes†.* Birth Defects Res A Clin Mol Teratol, 2011. **91**(S1): p. S2-S15.
24. Greenlees, R., et al., *Paper 6: EUROCAT member registries: organization and activities.* Birth Defects Res A Clin Mol Teratol, 2011. **91 Suppl 1**: p. S51-s100.
25. Kinsner-Ovaskainen, A., et al., *A sustainable solution for the activities of the European network for surveillance of congenital anomalies: EUROCAT as part of the EU Platform on Rare Diseases Registration.* European Journal of Medical Genetics, 2018. **61**(9): p. 513-517.
26. Dolk, H., et al., *Detection and investigation of temporal clusters of congenital anomaly in Europe: seven years of experience of the EUROCAT surveillance system.* Eur J Epidemiol, 2015. **30**(11): p. 1153-1164.
27. Loane, M., et al., *Paper 4: EUROCAT statistical monitoring: Identification and investigation of ten year trends of congenital anomalies in Europe.* Birth Defects Res A Clin Mol Teratol, 2011. **91**(S1): p. S31-S43.

For peer review only

28. Boulet, S.L., et al., *Feasibility of using the national hospital discharge survey to estimate the prevalence of selected birth defects.* Birth Defects Res A Clin Mol Teratol, 2006. **76**(11): p. 757-61.
29. Salemi, J.L., et al., *The Accuracy of Hospital Discharge Diagnosis Codes for Major Birth Defects: Evaluation of a Statewide Registry With Passive Case Ascertainment.* J Public Health Manag Pract, 2016. **22**(3): p. E9-e19.
30. Wang, Y., P.K. Cross, and C.M. Druschel, *Hospital discharge data: can it serve as the sole source of case ascertainment for population-based birth defects surveillance programs?* J Public Health Manag Pract, 2010. **16**(3): p. 245-51.
31. Andrade, S.E., et al., *Validity of health plan and birth certificate data for pregnancy research.* Pharmacoepidemiol Drug Saf, 2013. **22**(1): p. 7-15.
32. Frohnert, B.K., et al., *Validity of hospital discharge data for identifying infants with cardiac defects.* J Perinatol, 2005. **25**(11): p. 737-42.
33. Blais, L., et al., *Validity of congenital malformation diagnostic codes recorded in Québec's administrative databases.* Pharmacoepidemiol Drug Saf, 2013. **22**(8): p. 881-9.
34. Devine, S., *Validation of neural tube defects in the full featured--general practice research database.* Pharmacoepidemiol Drug Saf, 2008. **17**(5).
35. *EUROCAT Guide 1.4*. 14/02/2018]; Available from: http://www.eurocat-network.eu/aboutus/datacollection/guidelinesforregistration/guide1_4.
36. Garne, E., et al., *Paper 5: Surveillance of multiple congenital anomalies: Implementation of a computer algorithm in European registers for classification of cases.* Birth Defects Res A Clin Mol Teratol, 2011. **91**(S1): p. S44-S50.
37. Herskind, A.M., et al., *Comparison of Late Mortality Among Twins Versus Singletons With Congenital Heart Defects.* The American Journal of Cardiology, 2017. **119**(10): p. 1680-1686.
38. Gnanendran, L., et al., *Neurodevelopmental outcomes of preterm singletons, twins and higher-order gestations: a population-based cohort study.* Arch Dis Child Fetal Neonatal Ed, 2015. **100**(2): p. F106-F114.
39. Wang, W., et al., *Outcomes of Congenital Diaphragmatic Hernia in One of the Twins.* Am J Perinatol, 2019. **36**(12): p. 1304-1309.
40. Martinka, D., et al., *Respiratory morbidity in late preterm twin infants.* Arch Gynecol Obstet, 2019. **300**(2): p. 337-345.
41. Busby, A., et al., *Preventing neural tube defects in Europe: population based study.* BMJ, 2005. **330**(7491): p. 574-575.

Author contributions

JKM is the principal author and the project's Scientific Coordinator; ML is the Data Coordinator; EG is the Clinical Coordinator; JD, AN, AP, JR and HdW are Steering Committee and Standardisation Committee members with JKM, ML and EG; IB and ALB are Steering Committee members; AR is a Standardisation Committee member; JT is the senior statistician; JG the research fellow; HC the project manager. All authors contributed to, read and approved the final manuscript.

Collaborators

All below named members of the EUROlinkCAT Consortium have contributed to the development of the protocol with their knowledge and experience of the surveillance of congenital anomalies. Names are given in Consortium organisation numerical order:

For peer review only

Population Health Research Institute, St George's, University of London, UK: Abigail Reid, Liz Limb, Gillian Briggs, Nicholas Connor

Faculty of Life and Health Sciences, Ulster University, Northern Ireland, UK: Joanne Given

Hospital Lillebaelt, Region Syddanmark, Denmark and Section of Epidemiology, University of Copenhagen, Denmark: Stine Kjaer Urhoj, Mads Damkjær, Christina Neergaard Pedersen

Population Health Sciences Institute, Faculty of Medical Sciences, Newcastle University, Newcastle upon Tyne, UK: Svetlana V. Glinianaia

Emila Romagna Registry of Birth Defects (IMER), University of Ferrara, Italy: Gianni Astolfi, Annarita Armaroli; Neonatal Intensive Care Unit, IMER Registry, Department of Medical Sciences, University Hospital of Ferrara, Italy: Elia Ballardini; Territorial Care Service - Emilia Romagna Health Authority, Italy: Aurora Puccini

Klinika za dječje bolesti Zagreb, Croatia: Ljubica Boban

National Research Council-Institute of Clinical Physiology (Consiglio Nazionale delle Ricerche-Istituto di Fisiologia Clinica), Pisa, Italy: Michele Santoro, Alessio Coi, Silvia Baldacci, Lorena Mezzasalma

University of Groningen, University Medical Center Groningen, Department of Genetics, Groningen, the Netherlands: L. Renée Lutke, Nicole H.A. Siemensma-Mühlenberg

Public Health Wales National Health Service Trust, Wales, UK: David Tucker

Institut National de la Santé et de la Recherche Médicale, Paris, France: Babak Khoshnood, Nathalie Bertille, Nathalie Lelong, Makan Rahshenas

Rare Diseases Research Unit, Foundation for the Promotion of Health and Biomedical Research in the Valencian Region, Valencia, Spain; Public Health and Preventive Medicine Department, University of València, Spain; Public Health Regional Health Administration (DG Salud Publica y Adicciones), Generalitat Valenciana, Valencia, Spain: Clara Cavero Carbonell, Óscar Zurriaga, Laia Barrachina Bonet, Laura García Villodre

Uniwersytet Medyczny im. Karola Marcinkowskiego w Poznaniu, Poznań, Poland: Anna Jamry-Dziurla

Finnish Institute for Health and Welfare, Finland: Anna Heino, Sonja Kiuru-Kuhlefelt, Mika Gissler

OMNI-Net for Children International Charitable Fund, Rivne, Ukraine: Wladimir Wertelecki, Lyubov Yevtushok, Nataliia Zymak-Zakutnia, Diana Akhmedzhanova, Lyubov Ostapchuk, Oksana Tsizh, Serhii Lapchenko

Epidemiology Department, National Registry of Congenital Anomalies, National Institute of Health Doctor Ricardo Jorge: Carlos Matias Dias, Ausenda Machado, Ana João Santos, Liliana Antunes, Paula Braz

Register of Congenital Malformations, Centre Hospitalier Universitaire de La Réunion, Île de la Réunion, France: Hanitra Randrianaivo-Ranjatoelina, Benedicte Bertaut Nativel

Provinciaal Instituut voor Hygiëne (PIH), Antwerp, Belgium: Vera Nelen, Guy Thys, Elly Den Hond

Departamento de Salud Gobierno Vasco, Basque Country, Spain: Olatz Mokoroa Carollo

Swansea University, Wales, UK: Daniel Thayer, Ieuan Scanlon, Sue Jordan

Department of Global Public Health and Primary Care, University of Bergen, Bergen, Norway and Division of Mental and Physical Health Norwegian Institute of Public Health, Bergen, Norway: Kari Klungsøyr

Directorate for Health Information and Research, Malta: Miriam Gatt

For peer review only

Funding statement

This project has received funding from the European Union's Horizon 2020 Research and Innovation programme under grant agreement No 733001 for 5 years: 1st January 2017 to 31st December 2021.

Data statement

The standardised methods and common data models will all be available freely on the EUROlinkCAT website. Requests for collaboration to analyse data from the study dataset are encouraged and should be addressed to the EUROlinkCAT Management Team, by contacting JKM. Interactive results tables will be accessible on the EUROlinkCAT website (https://www.eurolinkcat.eu/) later in the study.

Competing interests statement

The authors declare that they have no competing interests.

Figure legends

Figure 1: Structure of mortality and EUROCAT data used for analyzing children's survival

Figure 2: Structure of hospital admissions, prescription data and EUROCAT data used for analyzing children's morbidity

For peer review only

Patient data - 1 row per EUROCAT case							
Child ID	Child date of Birth	Child date of death	Type Matching	Variables relating to linkage	Variables relating to child	Variables relating to mortality	Variables relating to mother

Figure 1: Structure of mortality and EUROCAT data used for analysing children's survival

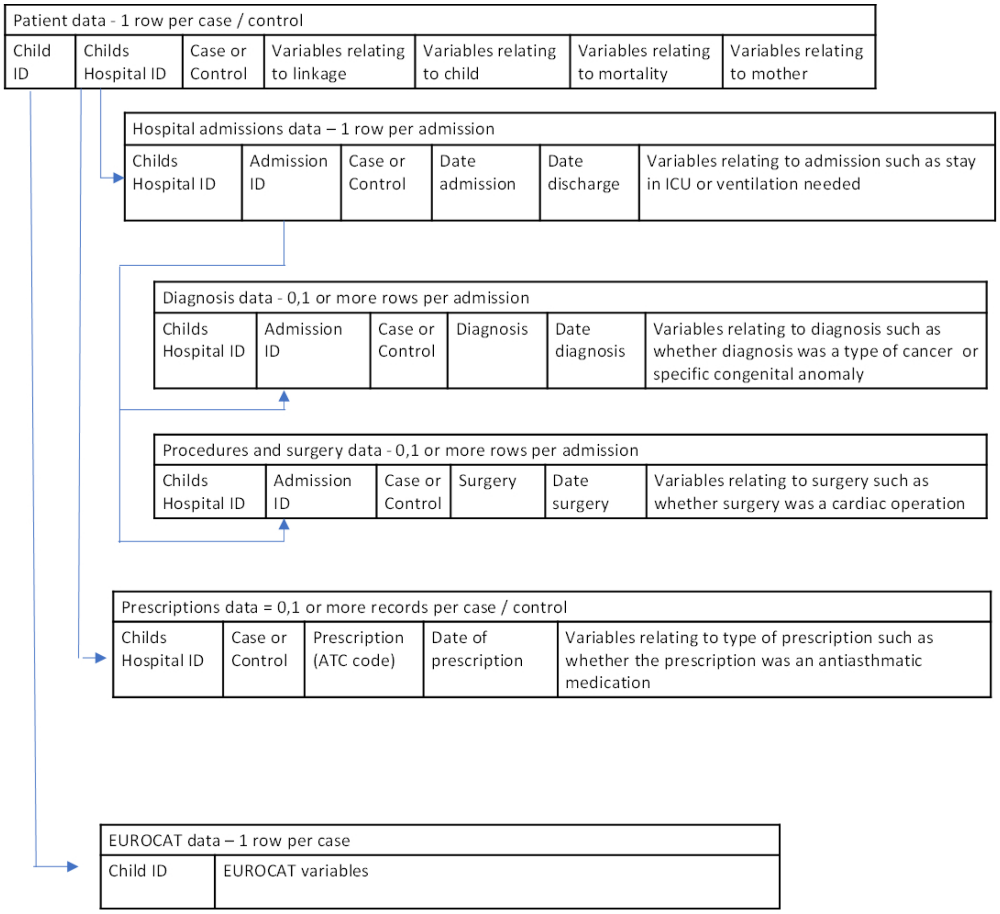

Figure 2: Structure of hospital admissions, prescription data and EUROCAT data used for analysing children's morbidity

Associated with document Ref. Ares(2016)3899547 - 25/07/2016

Proposal Evaluation Form

European Commission	**EUROPEAN COMMISSION**<br>Horizon 2020 - Research and Innovation Framework Programme	**Evaluation Summary Report - Research and innovation actions/Innovation actions**

Call: H2020-SC1-2016-RTD
Funding scheme: RIA
Proposal number: 733001
Proposal acronym: EUROlinkCAT
Duration (months): 60
Proposal title: EUROlinkCAT: Establishing a linked European Cohort of Children with Congenital Anomalies
Activity: SC1-PM-04-2016

N.	Proposer name	Country	Total Cost	%	Grant Requested	%
1	QUEEN MARY UNIVERSITY OF LONDON	UK	2,356,938	32.08%	2,356,938	32.08%
2	UNIVERSITY OF ULSTER	UK	1,402,544	19.09%	1,402,544	19.09%
3	REGION SYDDANMARK	DK	642,878	8.75%	642,878	8.75%
4	UNIVERSITY OF NEWCASTLE UPON TYNE	UK	558,875	7.61%	558,875	7.61%
5	UNIVERSITA DEGLI STUDI DI FERRARA	IT	343,218	4.67%	343,218	4.67%
6	Klinika za dječje bolesti Zagreb	HR	144,100	1.96%	144,100	1.96%
7	CONSIGLIO NAZIONALE DELLE RICERCHE	IT	200,462	2.73%	200,462	2.73%
8	ACADEMISCH ZIEKENHUIS GRONINGEN	NL	319,014	4.34%	319,014	4.34%
9	PUBLIC HEALTH WALES NATIONAL HEALTH SERVICE TRUST	UK	135,732	1.85%	135,732	1.85%
10	INSTITUT NATIONAL DE LA SANTE ET DE LA RECHERCHE MEDICALE (INSERM)	FR	26,282	0.36%	26,282	0.36%
11	FUNDACION PARA EL FOMENTO DE LA INVESTIGACION SANITARIA Y BIOMEDICA DELA COMUNITAT VALENCIANA	ES	116,838	1.59%	116,838	1.59%
12	UNIWERSYTET MEDYCZNY IM KAROLA MARCINKOWSKIEGO W POZNANIU	PL	138,775	1.89%	138,775	1.89%
13	TERVEYDEN JA HYVINVOINNIN LAITOS	FI	340,195	4.63%	340,195	4.63%
14	International Charitable Fund "OMNI-Net for Children"	UA	42,495	0.58%	42,495	0.58%
15	OTTO-VON-GUERICKE-UNIVERSITAET MAGDEBURG	DE	52,786	0.72%	52,786	0.72%
16	INSTITUTO NACIONAL DE SAUDE DR. RICARDO JORGE	PT	98,328	1.34%	98,328	1.34%
17	Centre Hospitalier Universitaire de La Réunion	FR	68,662	0.93%	68,662	0.93%
18	APB PIH	BE	50,582	0.69%	50,582	0.69%
19	ASOCIACION INSTITUTO BIODONOSTIA	ES	88,908	1.21%	88,908	1.21%
20	Biomedical Computing Limited	UK	138,072	1.88%	138,072	1.88%
21	Redburn Solutions Limited	UK	82,388	1.12%	82,388	1.12%
	Total:		7,348,073		7,348,073	

Abstract:
Over 130,000 children born in Europe every year will have a congenital anomaly (CA; birth defect). These CAs, which are often rare diseases, are a major cause of infant mortality, childhood morbidity and long-term disability. EUROCAT is an established European network of population-based registries for the epidemiologic surveillance of CAs. EUROlinkCAT will use the EUROCAT infrastructure to support 21 EUROCAT registries in 13 European countries to link their CA data to mortality, hospital discharge, prescription and educational databases. Each registry will send standard aggregate tables and analysis results to a Central Results Repository (CRR) thus respecting data security issues surrounding sensitive data. The CRR will contain standardised summary data and analyses on an estimated 200,000 children with a CA born from 1995 to 2014 up to age 10, enabling hypotheses on their health and education to be investigated at an EU level. This enhanced information will allow optimisation of personalised care and treatment decisions for children with rare CAs. Registries will be supported in using social media platforms to connect with families who live with CAs in their regions. A novel sustainable e-forum, "ConnectEpeople", will link these families with local, national and international registries and information resources. ConnectEpeople will involve these families in setting research priorities and ensuring a meaningful dissemination of results. Findings will provide evidence to inform national treatment guidelines, such as concerning screening programs, to optimise diagnosis, prevention and treatment for these children and reduce health inequalities in Europe. An economic evaluation of the hospitalisation costs associated with CA will be provided The CRR and associated documentation, including linkage and standardisation procedures and "ConnectEpeople" forum will be available post-EUROlinkCAT thus facilitating future local and EU level analyses.

Evaluation Summary Report

Evaluation Result

Total score: 15.00 (Threshold: 12)

Form information

SCORING

Scores must be in the range 0-5.

Interpretation of the score:

For peer review only - http://bmjopen.bmj.com/site/about/guidelines.xhtml

*0 The **proposal fails to address the criterion** or cannot be assessed due to missing or incomplete information.*

***1 Poor.** The criterion is inadequately addressed, or there are serious inherent weaknesses.*

***2 Fair.** The proposal broadly addresses the criterion, but there are significant weaknesses.*

***3 Good.** The proposal addresses the criterion well, but a number of shortcomings are present.*

***4 Very good.** The proposal addresses the criterion very well, but a small number of shortcomings are present.*

***5 Excellent.** The proposal successfully addresses all relevant aspects of the criterion.Any shortcomings are minor.*

Criterion 1 - Excellence

Score: **5.00** (Threshold: 4/5.00 , Weight: -)

The following aspects will be taken into account, to the extent that the proposed work corresponds to the topic description in the work programme:
Clarity and pertinence of the objectives
Soundness of the concept, and credibility of the proposed methodology
Extent that proposed work is beyond the state of the art, and demonstrates innovation potential (e.g. ground-breaking objectives, novel concepts and approaches, new products, services or business and organisational models)
Appropriate consideration of interdisciplinary approaches and, where relevant, use of stakeholder knowledge

EUROlinkCAT will exploit the European network of congenital anomaly registry (EUROCAT) infrastructure, seeking to link the data from these 21 registries (from 13 countries) to mortality, morbidity, hospital discharge, prescription and educational databases. This is a highly compelling application from an established network of institutions and investigators and the project builds on previous experience on data integration and harmonisation.

The concept of the proposed study is original and clear, and the proposed work is ambitious and achievable.

The proposal is timely; currently, limited information is available, especially in Europe, on the survival, health determinants, clinical course, hospitalisation costs, and educational achievements of children with congenital anomalies. The data on a very large number of children with congenital anomalies are impressive, with sizeable populations for rare disorders.

The project presents a unique opportunity to fill an important gap. Several data sources will be linked in several European countries to understand better the outcome and other aspects of congenital anomalies, which will inform families, health professionals and policy makers. The "ConnectEpeople" forum concept is novel, seeking more direct engagement with the parents of children with congenital anomalies.

The project plans to identify best practices for treatment options, evaluate the accuracy of existing electronic health care databases, and engage the relevant health authorities by establishing an Action Advisory Panel.

The proposal utilizes the appropriate interdisciplinary approaches and incorporates stakeholders' knowledge to understand better and improve clinical practice and health policy.

The potential problems with some of the data sources are not fully addressed.

Criterion 2 - Impact

Score: **5.00** (Threshold: 4/5.00 , Weight: -)

The following aspects will be taken into account:
The extent to which the outputs of the project would contribute to each of the expected impacts mentioned in the work programme under the relevant topic
Any substantial impacts not mentioned in the work programme, that would enhance innovation capacity, create new market opportunities, strengthen competitiveness and growth of companies, address issues related to climate change or the environment, or bring other important benefits for society
Quality of the proposed measures to:
- exploit and disseminate the project results (including management of IPR), and to manage research data where relevant
- communicate the project activities to different target audiences

The expected impacts including conceptual, methodological, analytical impacts of the project are well described. The project results will help inform policy and contribute to formulating best practice guidelines and improve clinical practice. The project will enable parents and patients to access pertinent information. The concept is well aligned with the impacts listed in the work programme.

The project can bring important benefits for society (evidence-based information, resource allocation, best clinical practice, socioeconomic research, policy change). Future work and programmes/registries can also benefit from the recommendations on coding standardisation but the panellists felt that potential impacts could have been addressed more explicitly with additional considerations.

The dissemination component of the proposal is adequately addressed with engagement of several stakeholders.

Facilitated by the Action Advisory Panel, the communication of project activities and findings to various target audiences are well summarised and liaisons are nominated to collaborate with relevant other networks and organisations.

Criterion 3 - Quality and efficiency of the implementation

Score: **5.00** (Threshold: 3/5.00 , Weight: -)

The following aspects will be taken into account:
Quality and effectiveness of the work plan, including extent to which the resources assigned to work packages are in line with their objectives and deliverables

For peer review only - http://bmjopen.bmj.com/site/about/guidelines.xhtml

Associated with document Ref. Ares(2016)3899547 - 25/07/2016

Appropriateness of the management structures and procedures, including risk and innovation management
Complementarity of the participants and extent to which the consortium as a whole brings together the necessary expertise
Appropriateness of the allocation of tasks, ensuring that all participants have a valid role and adequate resources in the project to fulfil that role

The project presents a coherent and effective work plan. The work plan is well distributed in WPs, and the description, deliverables, and milestones are well described. An adequate Gannt chart is included with timing of the different WPs.

Management structure and procedures are appropriate. Innovation and risk management (with risk mitigation measures) are adequately explained.

The consortium brings together partners with strong and relevant experience and expertise. The various partners have a long lasting collaborative history and show strong publication records, complementing each other in several areas.

The allocation of tasks is well planned and the resources requested are compatible with the work to be performed and appropriately allocated to the participants.

Gender differences are well described and fully taken into account in the study.

Scope of the proposal

Status: **Yes**

Comments (in case the proposal is out of scope)

Not provided

Operational Capacity

Status: **Operational Capacity: Yes**

If No, please list the concerned partner(s), the reasons for the rejection, and the requested amount.

Not provided

Exceptional funding of third country participants/international organisations

A third country participant/international organisation not listed in General Annex A to the Main Work Programme may exceptionally receive funding if their participation is essential for carrying out the project (for instance due to outstanding expertise, access to unique know-how, access to research infrastructure, access to particular geographical environments, possibility to involve key partners in emerging markets, access to data, etc.). (For more information, see the Online Manual)

Based on the information provided in the proposal, we consider that the following participant(s)/international organisation(s) that requested funding should exceptionally be funded:
(Please list the Name and acronym of the applicant, Reasons for exceptional funding and the Requested grant amount.)

Not provided

Based on the information provided in the proposal, we consider that the following participant(s)/international organisation(s) that requested funding should NOT be funded:
(Please list the Name and acronym of the applicant, Reasons for exceptional funding and the Requested grant amount.)

Not provided

Use of human embryonic stem cells (hESC)

Status: **No**

If yes, please state whether the use of hESC is, or is not, in your opinion, necessary to achieve the scientific objectives of the proposal and the reasons why. Alternatively, please also state if it cannot be assessed whether the use of hESC is necessary or not because of a lack of information.

Not provided

For peer review only - http://bmjopen.bmj.com/site/about/guidelines.xhtml

European Commission

PARTICIPANT PORTAL DIGITAL SEAL

This document is digitally sealed. The digital sealing mechanism uniquely binds the document to the modules of the Participant Portal of the European Commission, to the transaction for which it was generated and ensures its integrity and authenticity.

Any attempt to modify the content will lead to a breach of the electronic seal, which can be verified at any time by clicking on the digital seal validation symbol.

BMJ Open

EUROlinkCAT Protocol for a European population-based data linkage study investigating the survival, morbidity and education of children with congenital anomalies

Journal:	*BMJ Open*
Manuscript ID	bmjopen-2020-047859.R1
Article Type:	Protocol
Date Submitted by the Author:	04-May-2021
Complete List of Authors:	Morris, Joan; St George's University of London, Population Health Research Institute<br>Garne, Ester; Hospital Lillebaelt Kolding, Region Syddanmark<br>Loane , Maria ; Ulster University Faculty of Life and Health Sciences<br>Barisic, Ingeborg; Klinika za dječje bolesti Zagreb<br>Densem, James; Biomedical Computing Limited<br>Latos-Bieleńska, Anna; Uniwersytet Medyczny imienia Karola Marcinkowskiego w Poznaniu Wydzial I Lekarski<br>Neville, Amanda; University Hospital of Ferrara, IMER Registry (Emila Romagna Registry of Birth Defects)<br>Pierini, Anna; Institute of Clinical Physiology National Research Council<br>Rankin, Judith; Newcastle University, Population Health Sciences Institute<br>Rissmann, Anke; Otto von Guericke University Medical Faculty, Malformation Monitoring Centre Saxony-Anhalt<br>de Walle, Hermien; University Medical Centre Groningen, Department of Genetics<br>Tan, Joachim; St George's University of London, Population Health Research Institute<br>Given, Joanne; Ulster University Faculty of Life and Health Sciences<br>Claridge, Hugh; St George's University of London, Population Health Research Institute
**Primary Subject Heading**:	Paediatrics
Secondary Subject Heading:	Epidemiology
Keywords:	PAEDIATRICS, STATISTICS & RESEARCH METHODS, EPIDEMIOLOGY

SCHOLARONE™
Manuscripts

I, the Submitting Author has the right to grant and does grant on behalf of all authors of the Work (as defined in the below author licence), an exclusive licence and/or a non-exclusive licence for contributions from authors who are: i) UK Crown employees; ii) where BMJ has agreed a CC-BY licence shall apply, and/or iii) in accordance with the terms applicable for US Federal Government officers or employees acting as part of their official duties; on a worldwide, perpetual, irrevocable, royalty-free basis to BMJ Publishing Group Ltd ("BMJ") its licensees and where the relevant Journal is co-owned by BMJ to the co-owners of the Journal, to publish the Work in this journal and any other BMJ products and to exploit all rights, as set out in our licence.

The Submitting Author accepts and understands that any supply made under these terms is made by BMJ to the Submitting Author unless you are acting as an employee on behalf of your employer or a postgraduate student of an affiliated institution which is paying any applicable article publishing charge ("APC") for Open Access articles. Where the Submitting Author wishes to make the Work available on an Open Access basis (and intends to pay the relevant APC), the terms of reuse of such Open Access shall be governed by a Creative Commons licence – details of these licences and which Creative Commons licence will apply to this Work are set out in our licence referred to above.

Other than as permitted in any relevant BMJ Author's Self Archiving Policies, I confirm this Work has not been accepted for publication elsewhere, is not being considered for publication elsewhere and does not duplicate material already published. I confirm all authors consent to publication of this Work and authorise the granting of this licence.

For peer review only

EUROlinkCAT Protocol for a European population-based data linkage study investigating the survival, morbidity and education of children with congenital anomalies

Joan K Morris[1], Ester Garne[2], Maria Loane[3], Ingeborg Barišić[4], James Densem[5], Anna Latos-Bieleńska[6], Amanda Neville[7], Anna Pierini[8], Judith Rankin[9], Anke Rissmann[10], Hermien E.K. de Walle[11], Joachim Tan[1], Joanne Given[3], Hugh Claridge[1]

1. Population Health Research Institute, St George's, University of London, UK
2. Hospital Lillebaelt Kolding, Region Syddanmark, Denmark
3. Faculty of Life and Health Sciences, Ulster University, Northern Ireland, UK
4. Klinika za dječje bolesti Zagreb, Croatia
5. Biomedical Computing Limited, Battle, United Kingdom
6. Uniwersytet Medyczny im. Karola Marcinkowskiego w Poznaniu, Poznań, Poland
7. IMER Registry (Emila Romagna Registry of Birth Defects), University Hospital of Ferrara, Italy
8. National Research Council-Institute of Clinical Physiology (Consiglio Nazionale delle Ricerche-Istituto di Fisiologia Clinica), Pisa, Italy
9. Population Health Sciences Institute, Newcastle University, UK
10. Malformation Monitoring Centre Saxony-Anhalt, Medical Faculty Otto-von-Guericke-University Magdeburg, Magdeburg, Germany
11. University of Groningen, University Medical Center Groningen, Department of Genetics, Groningen, the Netherlands

Correspondence to Joan K Morris; jmorris@sgul.ac.uk

Word count: 5542
Keywords: Protocol; Congenital Anomalies; Linkage; Mortality; Morbidity

For peer review only

Abstract

Introduction

Congenital anomalies (CA) are a major cause of infant mortality, childhood morbidity and long-term disability. Over 130,000 children born in Europe every year will have a CA. This paper describes the EUROlinkCAT study, which is investigating the health and educational outcomes of children with CAs for the first 10 years of their lives.

Methods and analysis

EUROCAT is a European network of population-based registries for the epidemiological surveillance of CAs. EUROlinkCAT is using the EUROCAT infrastructure to support 22 EUROCAT registries in 14 countries to link their data on births with CAs to mortality, hospital discharge, prescription and educational databases. Once linked, each registry transforms their case data into a Common Data Model (CDM) format and they are then supplied with common STATA syntax scripts to analyse their data. The resulting aggregate tables and analysis results are submitted to a Central Results Repository (CRR) and meta-analyses are performed to summarise the results across all registries. The CRR currently contains data on 155,594 children with a CA followed up to age 10 from a population of 6 million births from 1995 to 2014.

Ethics

The CA registries have the required ethics permissions for routine surveillance and transmission of anonymised data to the EUROCAT central database. Each registry is responsible for applying for and obtaining additional ethics and other permissions required for their participation in EUROlinkCAT.

Dissemination

The CDM and associated documentation, including linkage and standardisation procedures, will be available post-EUROlinkCAT thus facilitating future local, national and EU-level analyses to improve health care. Recommendations to improve the accuracy of routinely collected data will be made. Findings will provide evidence to inform parents, health professionals, public health authorities and national treatment guidelines to optimise diagnosis, prevention and treatment for these children with a view to reducing health inequalities in Europe.

Article summary

Strengths and limitations of this study

- The implementation of a common data model enables the same centrally developed syntax script to be run in all registries which is efficient and ensures standardisation of analysis.

For peer review only

- The use of a reference population allows country differences to be adjusted for and enables more accurate comparisons of the burden of disease attributable to congenital anomalies across countries to be made.
- Merging births with congenital anomalies to their records in routine health care data enables a detailed evaluation of the accuracy of the data and enables improvements to be suggested.
- Being unable to share individual case data or aggregate data that might be disclosive reduces the power of the analysis that can be performed, particularly for rare anomalies.
- Only specific areas in Europe are represented, with a lack of data in particular from Eastern Europe.

For peer review only

Introduction

Over 130,000 children born in Europe every year will have a major CA, equivalent to 2.5% of all European births. CAs include structural defects, chromosomal anomalies and genetic syndromes. CAs are a leading cause of perinatal and infant mortality, especially in developed countries [1]. From 2003 to 2012, CAs were associated with about 40% of all infant deaths in Sweden and England [2]. There is a large variation in child death rates across Europe; in 2013 the child death rates (age 0-14 years) were 60% higher in the UK and Belgium compared to Sweden, with an additional 10 countries being 30% higher than Sweden [3]. To identify potentially preventable and remedial causes it is important to investigate the health inequalities in survival in children with CA across Europe.

It has been shown that relying on death certificates as a source of information on mortality due to CAs does not provide an accurate assessment of the survival for children with specific CAs. Death certificates state the direct or primary cause of death which may be infection, seizures or others and therefore may not mention the CA[4]. Copeland et al. (2007)[4] concluded that the only way to accurately study mortality and survival in children with rare CAs is to pool data across CA registries and link these to death registries.

Advances in fetal, neonatal and paediatric care have improved outcomes for individuals with some CAs, for example Down syndrome[5-7] and cardiac anomalies. Several studies have shown that children with CAs account for a very high proportion of all hospital admissions [8, 9]. However, there is a lack of information on the length of hospital stays for children with specific CAs, with most studies concerning children with Down syndrome, orofacial clefts or congenital heart defects (CHD) [10]. Often hospital stays are investigated for the first two or three years of a child's life[11-14]. However, Wehby et al. (2012) showed that hospital admissions for those born with orofacial clefts were increased at all ages up to 60 years of age[15]. Rarely has length of hospital stay been related to other factors, such as social class. Two studies (Derrington 2013 in the USA to 3 years of age and Hung 2011 in Taiwan for all ages) both identified other factors such as ethnicity and socio-economic factors as important influences on the length of inpatient stays in children and adults with Down syndrome[12, 16].

The proportion of children born with a CA surviving beyond infancy is increasing [5, 6]. How these children are performing in school and their additional educational needs is therefore becoming increasingly important as there may be a growing population of children and young people requiring additional support and resources in the future. However, apart from the more common genetic syndromes, there is a paucity of information about this[17, 18]. The American Heart Association reviewed the literature on children with CHD and concluded that they are at an increased risk of developmental delay, even once the frequent occurrence of genetic syndromes has been taken into account, particularly for neonates or infants requiring open heart surgery[19]. Wehby et al. (2015) also showed that children with isolated orofacial clefts were at a much greater risk of low achievement at school than their classmates[20]. A systematic review of neurocognitive outcomes following general anaesthesia and surgery in children concluded that exposure to general anaesthesia in young children did affect their development in some neurocognitive domains [21]. However, the authors recommended that the effects of surgery should be considered separately for each specific anomaly.

EUROCAT (https://eu-rd-platform.jrc.ec.europa.eu/eurocat) is a European network of population-based CA registries, which started in 1979 and increased to include 39 registries in 21 countries covering more than 29% of European births (1.7 million) per year [22-24]. The main objectives of EUROCAT are to provide essential epidemiologic information and surveillance on CAs in Europe, to evaluate the effectiveness of primary prevention and to assess the impact of developments in prenatal screening[25, 26]. Hence the emphasis is on information collected up to a baby's first year of life.

The aim of EUROlinkCAT is to investigate the survival, morbidity and educational outcomes of children with specific CAs for the first 10 years of their lives by linking births with CAs in EUROCAT registries to electronic health care and education databases. The availability of population-based data on births with CAs across the EUROCAT network will enable survival, morbidity and education to be investigated for specific CAs as well as differences in these outcomes across Europe according to specific risk factors and social inequalities to be explored.

Electronic health care data are increasingly being used by researchers to investigate the epidemiology of CAs, rather than using information from CA registries. Such health care data have often been found to be incomplete[27-31]. A small number of registries will analyse the maternal pregnancy records for women registered as having had a termination of pregnancy for a fetal anomaly (TOPFA) in EUROCAT. This will enable the accuracy of routine information on TOPFAs to be evaluated. The accuracy of CA coding in live births will be evaluated by comparing the EUROCAT data for live births with the CA diagnosis from the electronic health care databases covering both in-patient and out-patient visits. The information on death certificates will be compared to the anomalies recorded in EUROCAT. Recommendations will be developed to enable the maximum

For peer review only

information from electronic health care data to be extracted for research purposes and to quantify the amount of data that cannot be obtained.

This paper describes the design of the study, the methods used to obtain and analyse the linked data and evaluates the first three years' progress of EUROlinkCAT.

Methods and analysis

Design and Setting

In 2017, all EUROCAT registries were invited to participate in the EUROlinkCAT study. Twenty-two registries from 14 countries agreed to participate and to link all live births with a CA registered in their registries and born from 1st January 1995-31st December 2014. Almost all EUROCAT registries send anonymised data on CAs occurring in all livebirths, fetal deaths from 20 weeks gestation and TOPFAs to the EUROCAT central database. Comprehensive coding instructions [32] and the use of the EUROCAT Data Management Program (EDMP) to import data into the central database ensure that standard variables, definitions and coding are used by all registries in the network. CAs are coded locally using the WHO International Statistical Classification of Diseases and Related Health Problems (ICD) 9th or 10th Revision with the British Paediatric Association (BPA) code extension offering more specificity (Table 1). Cases with minor anomalies only are excluded (see EUROCAT Guide 1.4, Minor Anomalies for Exclusion (version 14.10.14)). Registries can code up to nine anomalies for each case and provide additional information in the specified text fields. Based on the ICD-9 or ICD-10 codes present, cases are automatically assigned by EDMP to defined major CA subgroups in accordance with the EUROCAT guide 1.4. A case with more than one major anomaly may be assigned to more than one subgroup. Since 2015, the central database has been hosted by the European Commission Joint Research Centre in Ispra (Italy).

Tables 1 and 2 provide a list of the 81 EUROlinkCAT CA subgroups which include structural anomalies, genetic syndromes and chromosomal anomalies that will be investigated. There are 60 EUROCAT subgroups (Table 1) and an additional 21 new CA subgroups not defined in EUROCAT (Table 2). The subgroups have been identified as being reasonably homogeneous to provide meaningful information and also to be prevalent enough to enable sufficiently precise estimates to be obtained from the analyses. For example, the EUROCAT subgroup "Chromosomal" was not included as it includes all genetic syndromes, but specific syndromes such as Down syndrome (a EUROCAT subgroup) and Di George syndrome (a new subgroup) were included. For some analysis, such as mortality, Down syndrome children will be analysed according to whether they have a cardiac anomaly and/or a gastrointestinal anomaly, as these are common and are likely to influence their survival. As outcomes are expected to be more severe for children with multiple and more complex CAs, analyses are also performed separately for children with isolated anomalies or with multiple anomalies defined according to the methodology by Garne et al.[33] . Isolated anomalies are defined as a CA in one organ system only or with a known sequence where multiple CAs cascade

For peer review only

as a consequence of a single primary anomaly. Multiple anomalies are defined as two or more major structural CAs in different organ systems, where the pattern of anomalies has not been recognised as part of a syndrome or sequence.

For peer review only

Table 1: EUROCAT congenital anomaly subgroups in EUROlinkCAT

EUROCAT Subgroups	ICD-10-BPA	ICD-9-BPA
**All anomalies ***	Q-chapter, D215, D821, D1810^, P350, P351, P371	74, 75, 27910, 2281^, 76076, 76280, 7710, 7711, 77121
**Structural anomalies**		
Spina Bifida	Q05	741
Hydrocephalus	Q03	7423
Severe microcephaly	Q02	7421
Congenital cataract	Q120	74332
Congenital Heart Defects (CHD)	Q20-Q26	745, 746, 7470-7474
Severe CHD	Q200, Q201, Q203, Q204, Q212, Q213, Q220, Q224, Q225, Q226, Q230, Q232, Q233, Q234, Q251, Q252, Q262	74500, 74510, 7452, 7453, 7456, 7461, 7462, 74600, 7463, 7465, 7466, 7467, 7471, 74720, 74742
Transposition of great vessels	Q203	74510
Ventricular Septal Defect	Q210	7454
ASD	Q211	7455
AVSD	Q212	7456
Tetralogy of Fallot	Q213	7452
Pulmonary valve stenosis	Q221	74601
Aortic valve atresia/stenosis	Q230	7463
Mitral valve anomalies	Q232, Q233	7465, 7466
Hypoplastic left heart	Q234	7467
Coarctation of aorta	Q251	7471
Patent ductus arteriosus (PDA) as only CHD in term infants (GA +37 weeks)	Q250	7470
Cystic adenomatous malformation of lung	Q3380	No code
Cleft lip with or without cleft palate	Q36, Q37	7491, 7492
Cleft palate	Q35	7490
Oesophageal atresia with/ without trachea-oesophageal fistula	Q390-Q391	75030-75031
Duodenal atresia or stenosis	Q410	75110
Atresia or stenosis of other parts of small intestine	Q411-Q418	75111-75112
Ano-rectal atresia and stenosis	Q420-Q423	75121-75124
Diaphragmatic hernia	Q790	75661
Gastroschisis	Q793	75671
Omphalocele	Q792	75670
Multicystic renal dysplasia	Q6140, Q6141	75316

For peer review only

Congenital hydronephrosis	Q620	75320
Hypospadias	Q54	75260
Limb reduction defects	Q71-Q73	7552-7554
Craniosynostosis	Q750	75600
**Chromosomal anomalies**		
Down syndrome	Q90	7580
Trisomy 13	Q914-Q917	7581
Trisomy 18	Q910-Q913	7582
Turner syndrome	Q96	75860, 75861, 75862, 75869
Klinefelter syndrome	Q980-Q984	7587
**Rare structural anomalies with a EUROCAT subgroup**		
Encephalocele	Q01	7420
Arhinencephaly / holoprosencephaly	Q041, Q042	74226
Anophthalmos / microphthalmos	Q110, Q111, Q112	7430, 7431
Anophthalmos	Q110, Q111	7430
Congenital glaucoma	Q150	74320
Anotia	Q160	74401
Common arterial truncus	Q200	74500
Double outlet right ventricle	Q201	No code
Single ventricle	Q204	7453
Triscuspid atresia and stenosis	Q224	7461
Ebstein's anomaly	Q225	7462
Pulmonary valve atresia	Q220	74600
Hypoplastic right heart	Q226	No code
Aortic atresia / interrupted aortic arch	Q252	74720
Total anomalous pulmonary venous return	Q262	74742
Choanal atresia	Q300	7480
Hirschsprung's disease	Q431	75130-75133
Atresia of bile ducts	Q442	75165
Annular pancreas	Q451	75172
Indeterminate sex	Q56	7527
Situs inversus	Q893	7593
VATER/VACTERL	Q8726	759895

For peer review only

* All Anomalies = ALL cases of congenital anomaly, excluding cases with only minor anomalies as defined in Section 3.2 in EUROCAT Guide 1.4 for cases born post-2005. Cases with more than one anomaly are only counted once in the "All Anomalies" subgroup.
^ ICD10 code D1810 (ICD 9 code 2281) is the code for cystic hygroma

Table 2: New congenital anomaly subgroups in EUROlinkCAT

**New subgroups for EUROlinkCAT**	**ICD-10-BPA**	**ICD-9-BPA†**
Structural anomalies		
Anomalies of corpus callosum	Q040	74221
Anomalies of intestinal fixation	Q433	7514
Unilateral renal agenesis	Q600	No code
Accessory kidney	Q630	75330
Bladder exstrophy	Q641	7535
Epispadia	Q640	75261
Posterior urethral valves	Q6420	75360
Prune Belly	Q794	75672
Arthrogryposis multiplex congenita	Q743	75580
Genetic syndromes		
Di George syndrome	D821	27910
Goldenhar syndrome	Q8704	75606
Cornelia de Lange syndrome	Q8712	759821
Noonan syndrome	Q8714	759896
Prader-Willi	Q8715	759872
Beckwith-Wiedemann syndrome	Q8730	759874
Williams syndrome	Q8784	No code
Angelman syndrome	Q8785	No code
Chromosomal anomalies		
Wolff-Hirschhorn syndrome	Q933	75832
Cri-du chat syndrome	Q934	75831
Karyotype XXX	Q970	75885
Sequences		
Pierre-Robin sequence	Q8708	75603

Linkage

Table 3 provides details of the linkages originally planned by the EUROCAT registries and the current linkages occurring (as of August 2020). The reasons why some registries could not link their data are

For peer review only

explored in detail in another paper submitted for publication - they include not being able to obtain the necessary permissions, relevant outcomes not being recorded in specific data sources and the time scale for the data supply being after the end of the study's funding. Currently, 19 registries are linking their data to mortality records, 15 plan to link to hospital in-patient records and seven to prescription records for the work package that will consider morbidity for children born with a CA. At the time of writing, nine registries plan to link their information on children with CAs to education records. To evaluate the accuracy of the routine health care data, five registries are additionally linking to out-patient data and four will also link to pregnancy information recorded in the mother's health records about TOPFAs. The 19 registries survey over 6 million births in the population.

Table 3: EUROCAT Congenital anomaly registries in EUROlinkCAT: start year, births in the population up to 2014, live births with an anomaly in the study period and ability to link to mortality, health care, prescription and education data

For peer review only

Congenital Anomaly Registry			Number of live births with an anomaly		Linkages Occurring (Y), Not occurring (N) and Planned but no longer occurring (¥)						
	Planned start year	Actual start year	Planned to be linked	Linked to mortality data by 8/2020	Mortality	Hospital Data for Child		Hospital Data for Mother		Prescriptions	Education
						In-patient	Out-patient	In-patient	Out-patient		
**Belgium: Antwerp**	1995	1997	8083	7865	Y	N	N	N	N	N	N
**Croatia: Zagreb**	1995	2011	2232	441†	¥	Y	¥	N	N		N
**Denmark: Funen**	1995	1995	2418	2425	Y	Y	Y	Y	Y	Y	Y
**Finland**	1995	1995	44869	42861	Y	Y	Y	Y	Y	Y	Y
**France: Île de la Réunion**	2002	NC	3855	NC	¥	¥	N	N	N	N	N
**France: Paris**	1997	1995	13335	11623	Y	N	N	N	N	N	N
**Germany: Saxony-Anhalt**	1995	2005	8821	8698	Y	N	N	N	N	N	N
**Italy: Emilia Romagna**	1995	2008	11447	7327	Y	Y	N	N	N	Y	Y
**Italy: Tuscany**	1995	2005	9827	5187	Y	Y	Y	N	N	Y	¥
**Malta**	1995	2005	2470	2718	Y	N	N	N	N	N	N
**Netherlands: Northern**	1995	2005	8567	8325	Y	Y	Y	Y	Y	Y	N
**Norway**	1999	1999	26938	27201	Y	N	N	N	N	N	N
**Portugal: South**	1995	2000*	3425*	2447*	N	Y	N	N	N	N	N
**Spain: Basque Country**	1995	1995	4883	5904	Y	¥	N	N	N	¥	N
**Spain: Valencian Region**	2007	2007	7438	7389	Y	Y	N	N	N	Y	N
**UK: East Midlands and South Yorkshire**	1998	AL	18549	AL	Y	Y	N	N	N	¥	Y
**UK: Northern**	2000	AL	8617	AL	Y	Y	N	N	N	¥	Y

**England**												
**UK: South West England**	2005	AL	11671	AL	Y	Y	N	N	N	¥	Y
**UK: Thames Valley**	1995	AL	5142	AL	Y	Y	N	N	N	¥	Y
**UK: Wales**	1998	1998	18239	18128	Y	Y	Y	Y	Y	Y	Y
**UK: Wessex**	1995	AL	7771	AL	Y	Y	N	N	N	¥	Y
**Ukraine: West**	2005	2006	6166	5835	Y	Y	N	N	N	N	N
**Total**			234763	155594†	19	15	5	4	4	7	9

¥: Linkages are no longer planned as of August 2020
†: The 441 cases in Zagreb were not included in analyses due to poor quality of the mortality linkage
The registry in Basque was unable to complete the planned linkages to hospital data due to COVID-19
NC: Linkages could not be completed
AL: Awaiting linkage as of August 2020
***: Values for WP4 morbidity linkage have been provided, as mortality linkage was never planned**

For peer review only

For the evaluation of survival and morbidity, each child will be followed-up for a maximum of ten years. This age cut-off has been chosen to enable enough children to be identified and followed-up; a longer follow-up would mean fewer children would be eligible as currently national or local electronic health care record sources often do not go back more than 10 years. For education the maximum follow-up is until the end of compulsory school age (typically 16 across participating countries), although for some registers data are only available for a shorter period of follow-up. The longer follow-up was chosen in Finland where there are no national education tests and national data on education attainment are available at the age of 15-16 (9th grade).

Reference Population

Where possible, each registry obtained information from electronic health care records, prescription records and education records on children without a CA. The definition of these "control" cohorts will vary according to the registry, ranging from all children in the same population covered by the registry to a 10% random sample of children stratified by birth year and child's sex. The use of such a reference population is essential in interpreting differences across countries, as it will provide information on key outcomes, such as duration of hospital stays and medication prescribing, on children without reported anomalies, which is expected to vary by country. Table 4 provides information on the reference populations being identified.

Table 4: Use of a reference population in morbidity and education analyses

**Congenital Anomaly Registry**	**Reference Population**
Croatia: Zagreb	Sample of children
Denmark: Funen	Whole Population
Finland	Whole Population
France: Île de la Réunion	Not provided
Italy: Emilia Romagna	Whole Population
Italy: Tuscany	10% of population
Netherlands: Northern	10% of Population
Portugal: South	Sample of children
Spain: Basque	Not provided
Spain: Valencian Region	Whole Population
UK: East Midlands and South Yorkshire	Aggregate data from population for morbidity and population sample for Education
UK: Northern England	Aggregate data from population for morbidity and population sample for Education
UK: South West England	Aggregate data from population for morbidity and population sample for Education
UK: Thames Valley	Aggregate data from population for morbidity and population sample for Education
UK: Wales	Whole Population
UK: Wessex	Aggregate data from population for morbidity and population sample for Education

For peer review only

Ukraine: West	No longer in morbidity study

Standardisation and Common Data Models

EUROCAT registries submit 96 core and non-core variables to the EUROCAT central database providing pseudonymised information on the baby and mother, diagnosis, karyotype (if known), exposure, family history, and socio-demographic details. These have already been standardised and Table 5 lists the 52 variables of which 34 are core variables and their common coding scheme that are used in the EUROlinkCAT study. In contrast, all the data obtained from linkage have to be standardised to a common format, as the health care and educational systems across Europe use different native languages and coding classification schemes. To do this, each registry provided their data dictionaries describing the variables in their local databases including the variable names, format, definitions, and coding schemes.

Table 5: Standardised variables from the EUROCAT database

	**EDMP Variables used (core variables are shaded in blue)**	
**Baby and Mother**		
1	CENTRE	Centre Number
2	NUMLOC	Local ID of case
3	BIRTH_DATE	Date of Birth
4	SEX	Sex
5	NBRBABY	Number of babies delivered
6	SP_TWIN	Specify twin type of birth, like or unlike, zygosity
7	NBRMALF	Number of malformed in multiple set
8	TYPE	Type of birth
9	CIVREG	Civil registration status
10	WEIGHT	Birth weight
11	GESTLENGTH	Length of gestation in completed weeks
12	SURVIVAL	Survival beyond one week of age
13	DEATH_DATE	Date of death
14	DATEMO	Date of birth of mother
15	AGEMO	Age of mother at delivery
16	BMI	Maternal Body Mass Index
17	RESIDMO	Mother's residence code
**Diagnosis**		
19	WHENDISC	When discovered
20	CONDISC	Condition at discovery
21	AGEDISC	If prenatally diagnosed, gestational age at discovery
22	FIRST PRE	First positive prenatal test
24	KARYO	Karyotype of infant/fetus
25	SP_KARYO	Specify karyotype
26*	GENTEST	Genetic Test
27*	SP_GENTEST	Specify genetic test
28	PM	Post mortem examination
29	SURGERY	First surgery for malformation performed or planned
30	SYNDROME	Syndrome
31	SP_SYNDROME	Specify Syndrome
32	MALFO1	malformation
33	SP_MALFO1	Specify malformation
34	MALFO2	As MALFO1
35	SP_MALFO2	Specify malformation
36	MALFO3	As MALFO1
37	SP_MALFO3	Specify malformation

38	MALFO4	As MALFO1
39	SP_MALFO4	Specify malformation
40	MALFO5	As MALFO1
41	SP_MALFO5	Specify malformation
42	MALFO6	As MALFO1
43	SP_MALFO6	Specify malformation
44	MALFO7	As MALFO1
45	SP_MALFO7	Specify malformation
46	MALFO8	As MALFO1
47	SP_MALFO8	Specify malformation
57	OMIM	OMIM code / Type of Mendelian Inheritance
**Exposure and family history**		
58	ASSCONCEPT	Assisted conception (where available)
59##	OCCUPMO	Mother's occupation at time of conception
**Sociodemographic**		
91	MATEDU	Maternal education
92*	SOCM	Socioeconomic status of mother
93 *	SOCF	Socioeconomic status of father
94	MIGRANT	Migrant status
**Derived variables**		
	Byear	Year of birth
	birth_type	Livebirth, Stillbirth, Spontaneous abortion, TOPFA, Not known Definitions of stillbirths and spontaneous abortions vary between regions. This variable recodes birth type according to EUROCAT's specifications: cases with gestational age ≥ 20 weeks are re-coded as "stillbirths" (irrespective of the local definition of stillbirth/spontaneous abortion).
	casestatus	Only cases with casestatus = 1 or 2
	al1-al114	EUROCAT subgroups: (0 = No, 1 = Yes). Based on EUROCAT coding in Guide 1.4
	mult_malf	Algorithm for case classification into isolated and multiple anomalies

EUROCAT Guide 1.4 use ISCO-08 classifications

* See work of the standardisation committee (viii)

Table 6 shows how the variable sex (male and female) is coded for children in the different registries with different variable names, different formats and different coding schemes. For each sub-study in EUROlinkCAT, a common data model (CDM) containing all variables required for its analyses was developed. All the EUROlinkCAT CDMs contain the variable L_CH_SEX, defined as "sex of child" and with a coding scheme in integer format of 1=male, 2=female, 3=Indeterminate, 9=Not known '.'= Not recorded or not available for study. Ulster University (UU) used the information in each registry's data dictionary to create the new EUROlinkCAT "standardised" L_CH_SEX variable. UU, in collaboration with the registries, created registry-specific syntax scripts to standardise all the variables in the EUROlinkCAT CDMs. Appendices 1, 2 and 3 list all the variables included in the sub-studies (mortality, electronic health care records and prescription records).

Table 6: Coding of male or female of the live births in different linked databases in EUROlinkCAT

Code

For peer review only

Centre	Variable name	Male	Female
UK: Wales	DEC_SEX_CD	1	2
Germany: Saxony-Anhalt	EF306	1	2
Finland	SUKUP	1	2
Italy: Tuscany	SESSO	1	2
France: Île de la Réunion	SexeDefunt	1	2
France: Paris	SexeDefunt	1	2
Netherlands: Northern	geslacht	1	2
Croatia: Zagreb	GENDER	M or 1	F or 2
Ukraine: West	CH_SEX	1	2
Belgium: Antwerp	SEX	1	2
Norway	KJONN	1	2
UK: England	sex	1	2
Spain: Basque Country	SEXO	1	6
Spain: Valencian Region	SEXO	1	6
Malta	gender	M	F
Italy: Emilia Romagna	SEX	M	F
Denmark: Funen	C_SEX	M	K

The CDMs also specify how the data are stored. For mortality, all the relevant variables occur only once for each EUROCAT case and are stored in the same data file (or table) (Figure 1). However, when analysing hospital admissions, each child may have more than one admission and for each admission may receive more than one diagnosis. Therefore, the hospital admissions data are stored in a separate data file (or table) from the diagnoses data and separately from the EUROCAT data on the child; each data file contains a reference key which serves to link all records belonging to one person for analysis (see Figure 2). The standardisation syntax scripts from UU specify the separate data files (or tables) for each linkage containing all the variables in the CDMs.

UU included validation routines in the syntax scripts to determine if the data have been correctly transformed. For example, it is checked that a date of death does not occur prior to the date of birth; whilst its primary purpose is to ensure data had been standardised correctly, it can also reveal any errors in the linked data.

A CDM is not being defined for the linked education data as there is limited scope for comparison and pooling of data across countries. This is due in part to inherent differences in the educational

For peer review only

stages and systems, the variability in data available, and fewer registries being able to participate (5 of the registries are from England, and 1 from Wales, Denmark, Italy and Finland).

Work of the Standardisation Committee

In addition to defining the CDM and its structure, the EUROlinkCAT Standardisation Committee was responsible for taking other decisions, usually in consideration of local data characteristics, to ensure that data were meaningful and comparable across registries. The most important issues are listed below:

i. **Inclusion according to gestational age (GA) at birth**: For the mortality study all livebirths with a GA below 24 weeks were excluded, as these cases could have been miscoded terminations of pregnancy with signs of life at birth. However, after running the mortality analysis, it was noticed locally that there were survivors in EUROCAT registries at GA 23 weeks. For the morbidity studies, the exclusion criterion was lowered to be below 23 weeks.

ii. **Strength of linkage**: The success of data linkage depended on the matching method and type of personal identifiers used. Where a national unique identifier (ID) was available (e.g. Denmark, Finland) over 99% of cases were matched, but success rates were generally lower when intermediary databases and a combination of other identifiers (e.g. names, postcodes) were required to establish a match, particularly if these were incomplete or incorrect. A standard way of evaluating confidence in a match had to be developed so that decisions on inclusion for analyses could be consistently made, in order to avoid bias.

iii. **Gestational age groups**: The gestational age at birth was categorised into <28 weeks, 28-31 weeks, 32-36 weeks and 37+ weeks when analysing survival, but due to small numbers of survivors at under 28 weeks gestation, the two lowest gestational age categories were combined to <32 weeks.

iv. **Birth weight**: Birth weight was categorised into very low birth weight '<1500 g', low birth weight '≥1500 g to <2500 g', normal birth weight '≥2500 g to 3999 g' and high birth weight `≥4000 g'. Births <1000 g were not distinguished from those between 1000-1499 g as there were too few cases for the data to be analysed accurately.

v. **Singletons vs. multiples**: There is uncertainty about whether the survival in twins with CAs is lower than that in singletons [34-37]. Hence, all survival analyses were performed on singletons alone and then multiples and singletons combined (multiples were not analysed alone as for many registries small numbers would limit the analyses that could be performed). This enables the survival of singletons and multiples in children with CAs to be analysed in detail. When examining morbidity, multiplicity was treated as one of the risk factors for increased risk of hospitalisations and lengths of stay to enable any association to be analysed, but with less detail than for survival. The majority of analyses included singletons and multiples combined.

For peer review only

vi. **Prenatal diagnoses**: The gestational age at prenatal diagnoses was categorised into <22 weeks, 22-31 weeks, 32+ weeks, gestational age not known and no prenatal diagnosis. For Finland, the gestational age was often not recorded, only the trimester of diagnoses. Finland's first trimester diagnoses (week 0 to week 12) mapped exactly to the EUROlinkCAT <22 weeks category. It was decided that Finland's 2nd trimester (week 13 to week 27) diagnoses were assumed to occur at 22-31 weeks and 3rd trimester (week 28 onwards) diagnoses occurred at 32+ weeks. These assumptions were also checked based on the distribution of those cases in Finland with a "known" age at discovery and the assumptions held.

vii. **Length of stay**: The length of stay (LOS) of the child in hospital was calculated after excluding the stay associated with the birth. Methods of identifying the birth stay varied in different countries. For hospital admissions where admission and discharge occurred on the same day, the LOS was considered to be 0.5 days. If an admission record was missing a discharge date, then discharge date = date of admission + 2*(date of latest procedure – date of admission). The date of discharge was set to the date of the child's 10th birthday or the end of the study period if it was after either of these two dates.

viii. **Socio-economic status (SES)**: All registries had different variables that could be considered to be a measure of the mother's SES. The variables included maternal occupation, maternal education and index of multiple deprivation derived from residential codes at birth. Registries were asked to select the variable they believed was the most relevant and to re-code their selected SES proxy variable into three groups of approximately equal proportion to enable comparing between, for example, mothers in the highest group to mothers in the lowest group. The effect of SES on survival would be analysed using Cox proportional hazard models within each registry. However, only 7 registries were able to provide a proxy SES variable that was reasonably complete for some or all of the time period of the study. It was also planned to investigate the association between risk factors such as birth weight after adjusting for SES, but due to the lack of information on SES this was not included in further multivariable analyses.

ix. **Maternal country of birth**: It was determined that the maternal country of birth variable would be used as a proxy for non-European ethnic origin, as we were aware that ethnic origin is poorly recorded. However, for those registries with reasonably complete data on this, almost 100% of children were reported as being of European ethnic origin. Therefore, this variable was not included in subsequent analyses as the number of children considered as "Non-European ethnic origin" was too small to analyse.

x. **Cause of death:** cause of death based on the death certificates was classified for deaths <1 year and for 1-9 years separately. Death related to preterm birth is very common in the first year after birth, but not as relevant to children at 1-9 years of age. Injuries and poisoning are more common after the first year. The main causes of deaths were classified into 6 groups for deaths <1 year and 11 groups for children aged 1-9 years. When working with the results tables it was clear that many of these classification groups included many small numbers and data could not be extracted from the

For peer review only

databases. For some registries it was only possible to give cause of death as either "congenital anomaly" or else "any other cause of death".

xi. **Surgery**: A number of different coding systems were used across registries to code surgeries and other procedures (e.g. NCSP by NOMESCO, ICD-9-CM, OPCS-4). Frequency lists for all codes describing surgeries and other procedures were obtained from the linked datasets. Two paediatricians then independently determined if a code was a surgery or for another procedure and then a consensus between the two clinicians was reached over codes classified as codes for surgeries. Further sub-division into anomaly-specific surgeries was carried out for anomalies for which specific surgeries could be identified that would be expected to be performed on these children.

xii. **Intensive care**: It was planned to analyse the number of days in intensive care, however only five registries could provide this. Therefore, only whether a child had ever been admitted to intensive care was analysed rather than their length of stay.

xiii. **Ventilation**: It was planned to analyse the number of days on ventilation. However, as it was decided that the length of stay in intensive care was not going to be analysed, the same decision was made for ventilation and only whether a child had ever been on ventilation was analysed.

Assessment of Quality of Linkage and Quality of Linked Data

Many registries linked their data to National Vital Statistics, which are databases that record all live births with follow up until the child dies or emigrates outside the country/region of interest. Therefore, for these registries for the survival analysis, any child whose record was not in the National Vital Statistics Database was judged to be a non-match and overall linkage could be assessed. Some registries were only able to link to death certificates which meant that a non-match, i.e. no death certificate found, was assumed to indicate that the child was still alive. The data from these registries were only included in the survival analyses if there was additional information about the quality of the linkage. For example, in Malta, due to the small well-defined population, there was confidence that all deaths had been identified.

In some countries all national databases use the same unique ID number (for example Finland). So, identifying a child in the National Vital Statistics meant that there was confidence that any hospital stays up to ten years of age would also be identified. For other registries, as not all children were likely to be admitted to hospital, each case was searched for in, not only the in-patient hospital database (which included the mothers visit for the birth), but also any other health care databases (such as out-patient, primary-care or prescription databases) for longer than the ten years of follow-up in the study. The lack of information in any health care database was judged to mean a non-match. Sensitivity analysis was performed to assess if there were differences in results if the non-matches were included.

For peer review only

For the education data, all children known to be alive were assumed to be included in the National Education Databases and therefore any case not identified was assumed to be a non-match. Syntax scripts were developed centrally by St George's, University of London (SGUL) to evaluate the accuracy of the linkage and identify any factors leading to missed links (for example deaths within the first week of life). For each registry, the proportion of births in any single year of data that have not been linked is calculated and the data from any year with less than 85% of cases linked will be excluded from further analyses. Secondly, the quality of linked data items was also evaluated: a variable that was >20% missing in a year would be excluded from any analysis in which it featured; and variables that were recorded by both the CA registry and the linked database would be compared for agreement, by year. In general, it was found that data quality was poorer in the earlier years and tended to improve over time; however if data quality fluctuated across the years, then only the longest consecutive period where quality was above the threshold would be analysed.

Statistical Analysis

Protocols and syntax scripts are developed centrally to create aggregate data and perform specific analyses on the individual cases in each standardised data set in STATA (Version 13 and upwards). This allows each register to submit aggregated data and analytical results (e.g. Kaplan-Meier estimates, hazard ratios and confidence intervals), rather than individual case data, to the EUROlinkCAT CRR at UU, UK using a secure web platform. UU collates the aggregate data and results and provides these data to the researchers responsible for the different analyses and publications. Multi-centre European analyses will be performed by combining the individual registries' aggregated data and analytic results, using meta-analytic techniques. Additional work is required to develop suitable models for combining survival data from several registries when the sample sizes are very small as observed in many registries.

Small Number Restrictions (Statistical Disclosure Control)

Four countries have limitations on the release of aggregate data and analytic results if the numbers of births involved are very small (generally under eight births). This situation arises in many analyses involving specific CAs, as CAs are rare, with some affecting less than 1 in 10,000 livebirths. Solutions to enable the maximum amount of data to be included in all multi-centre European analyses varied according to country. The Northern Netherlands released data if all exported results were rounded to the nearest 5. Rounding all frequencies ensures that original numbers cannot be inferred. For Denmark, a few named researchers at SGUL and UU were allowed access to the aggregate data for the purpose of collating and including in pooled-analysis, on condition that it was securely stored and processed; that any individual results involving fewer than five people were not released; and that personal identification was not possible from any released results. The SAIL databank (Wales) provided data to the CRR with the requirement that aggregate data on fewer than five people were

For peer review only

not released and could not be calculated from any information in the public domain. The registry from Antwerp, Belgium could not release any information on three or fewer cases.

Patient and Public Involvement

A series of focus groups has been held in different European countries involving parents with a child with one of four pre-defined CAs with different health problems covering learning difficulties, physical disabilities, visible defects and non-visible defects with higher mortality. The four anomalies selected were: CHD requiring surgery (referred to as severe CHD – a usually non-visible defect with high mortality), cleft lip (CL; a visible defect often with speech problems), spina bifida (SB; a physical disability with associated incontinence problems) and Down syndrome (DS; Trisomy 21; a visible defect with learning difficulties and often associated with CHD). The focus groups have investigated parental experiences of having a child with one of the above anomalies and assessed parental research priorities ADD when paper published.

In addition, a European survey concerning the diagnosis, medical care, education and everyday life will be distributed to parents across Europe with children with the same four CAs as described above. Registries will ensure the questions in the survey are appropriate for their country (for example the provision of health services, given how this differs in various European countries) and will translate the survey into their native language with back translation to confirm the accuracy of the translation. The aim is for the survey to be distributed via social media by parent support groups across Europe to engage with a wide spectrum of parents.

Discussion

The aggregate data and results from the CRR in EUROlinkCAT will provide important information on the survival, morbidity and education of children born with a CA in Europe. Researchers in each CA registry will be encouraged to also perform specific local analysis, in order to fully exploit the research potential of linked datasets. The establishment of a method of standardising data from each registry linkage into a CDM provides valuable infrastructure enabling future multi-national studies to be performed in an efficient manner and new registries to become involved.

The strength of this study is that the researchers are a multidisciplinary group, many of whom have collaborated successfully for many years through being members of EUROCAT. In addition, EUROlinkCAT is able to build on all the standardisation procedures already established in EUROCAT. The implementation of a CDM enables the same centrally developed syntax script to be run in all the different registries which is efficient and also ensures standardisation of analysis across the registries. The use of a reference population when analysing health care data will aid in the identification of the source of differences between registries (for instance average LOS in hospitals will differ) and therefore enable us to better quantify the burden of disease attributable to CAs in

For peer review only

each country. Comparisons of the accuracy of health care databases with respect to recording CA cases will be informative and enable improvements in those areas with less accurate data. We will also be developing recommendations on how to use the available health care data in an optimal way to provide information on children with CAs in areas without active CA registries.

One of the challenges of EUROlinkCAT is the ability of the CA registries to link their data to external data sources due to different local data information governance issues and the availability of suitable electronic health care databases. This requires flexibility in including registries in only specific sub-projects and acceptance that not all registries may be able to perform the linkages planned. In addition, some registries require support from other partners in all aspects of the project, including applying for ethics permissions, adapting protocols, standardising data and running statistical syntax scripts. The restriction of not being able to share individual case data and also aggregate data that might be disclosive or identifiable means that all analyses must be performed locally using a generic modelling strategy. This does limit the use of iterative procedures to explore data in detail. The major limitation to the study is that only specific areas in Europe are represented, with a lack of data in particular from Eastern Europe. Interpretation of differences across Europe is challenging as it will be essential to interpret results in the light of knowledge about the differences in health care and education practices across Europe.

The EUROlinkCAT project will enable important hypotheses concerning the survival, health and education of children with CAs in Europe to be investigated. The standardised methods and CDMs will all be available freely on the EUROlinkCAT website and will be available for use in future research projects to benefit from and build on this work, so as to enable other multi-centre European projects to exploit routine health care data available in Europe.

Ethics and dissemination

The CA registries have the required ethics permissions and procedures for routine surveillance, data collection and transmission of anonymised data to the EUROCAT central database, according to national guidelines and they were required to submit evidence of these permissions to the EUROlinkCAT ethics portfolio. Local registries follow national legislation as to whether parental consent is needed for registration of babies with anomalies. Each registry was responsible for applying for and obtaining the additional ethics and other permissions (e.g. data sharing agreements) required to link and analyse their data for EUROlinkCAT. This was an extremely lengthy process in some countries as the original data collection did not include expectation or consent for the data to be used in research, and a new legal basis had to be established. Additional assurances and procedures were adopted by registries (e.g. publication of privacy notices) to ensure compliance with the General Data Protection Regulation (GDPR) which came into force on 25 April 2018 in EU countries. A checklist of minimum specifications for data storage/backup was completed by each registry. Three registries took over three years to get the ethics, legal basis, data protection,

For peer review only

information governance and data sharing agreements in place. UU obtained ethics permission for the CRR.

Each registry participating in the focus groups with parents was responsible for ensuring the correct ethics approvals were in place. Similarly, the registries participating in the dissemination of the parents' survey will be responsible for ensuring the necessary ethics permissions are obtained.

An Ethics and Data Protection Advisory Board (EDPB) consisting of three independent advisors with the relevant expertise monitor all ethical considerations in this project.

The CRR will be used for multiple studies and the results from these will be disseminated in peer reviewed papers and conference presentations. It is hoped that the experience gained with distributing the parents' survey using parent support groups and social media will also lead to development of a framework to enable dissemination of results to be made more directly to parents. In addition, a series of reports will be written including recommendations for improving the collection and analysis of data on CAs in routinely collected data in the health care databases.

For peer review only

References

1. Boyle, B., et al., *Estimating Global Burden of Disease due to congenital anomaly: an analysis of European data.* Arch Dis Child Fetal Neonatal Ed, 2018. **103**(1): p. F22-f28.
2. Zylbersztejn, A., et al., *Child mortality in England compared with Sweden: a birth cohort study.* Lancet, 2018. **391**(10134): p. 2008-2018.
3. Wang, H., et al., *Global, regional, and national levels of neonatal, infant, and under-5 mortality during 1990-2013: a systematic analysis for the Global Burden of Disease Study 2013.* Lancet, 2014. **384**(9947): p. 957-79.
4. Copeland, G.E. and R.S. Kirby, *Using birth defects registry data to evaluate infant and childhood mortality associated with birth defects: An alternative to traditional mortality assessment using underlying cause of death statistics.* Birth Defects Res A Clin Mol Teratol, 2007. **79**(11): p. 792-797.
5. Wu, J. and J.K. Morris, *The population prevalence of Down's syndrome in England and Wales in 2011.* Eur J Hum Genet, 2013. **21**(9): p. 1016-9.
6. Tennant, P.W., et al., *20-year survival of children born with congenital anomalies: a population-based study.* Lancet, 2010. **375**(9715): p. 649-56.
7. Brodwall, K., et al., *The five-year survival of children with Down syndrome in Norway 1994–2009 differed by associated congenital heart defects and extracardiac malformations.* Acta Paediatrica, 2018. **107**(5): p. 845-853.
8. Muranjan, M. and P. Vijayalakshmi, *The Unforeseen Toll of Birth Defects and their Economic Burden at a Tertiary Care Public Institute in Mumbai.* Ind J Pediatr, 2014. **81**(10): p. 1005-1009.
9. Simeone, R.M., et al., *Population-based study of hospital costs for hospitalizations of infants, children, and adults with a congenital heart defect, Arkansas 2006 to 2011.* Birth Defects Res A Clin Mol Teratol, 2015. **103**(9): p. 814-20.
10. Shetty, S., et al., *Length of stay and cost analysis of neonates undergoing surgery at a tertiary neonatal unit in England.* Ann R Coll Surg Engl, 2016. **98**(1): p. 56-60.

11. Fitzsimons, K.J., et al., *Hospital care of children with a cleft in England.* Arch Dis Child, 2013. **98**(12): p. 970-974.
12. Derrington, T.M., et al., *Racial/ethnic differences in hospital use and cost among a statewide population of children with Down syndrome.* Res Dev Disabil, 2013. **34**(10): p. 3276-87.
13. Dawson, A.L., et al., *Hospitalizations and associated costs in a population-based study of children with Down syndrome born in Florida.* Birth Defects Res A Clin Mol Teratol, 2014. **100**(11): p. 826-36.
14. Hook-Dufresne, D.M., et al., *The economic burden of gastroschisis: costs of a birth defect.* J Surg Res, 2015. **195**(1): p. 16-20.
15. Wehby, G.L., et al., *The effects of oral clefts on hospital use throughout the lifespan.* BMC Health Services Research, 2012. **12**(1): p. 58.
16. Hung, W.J., et al., *Cost of hospitalization and length of stay in people with Down syndrome: evidence from a national hospital discharge claims database.* Res Dev Disabil, 2011. **32**(5): p. 1709-13.
17. O'Toole, C., et al., *Parent-mediated interventions for promoting communication and language development in young children with Down syndrome.* The Cochrane database of systematic reviews, 2018. **10**(10): p. CD012089-CD012089.
18. Iyer, N.P., et al., *Outcome of fetuses with Turner syndrome: a 10-year congenital anomaly register based study.* J Matern Fetal Neonatal Med, 2012. **25**(1): p. 68-73.
19. Marino, B.S., et al., *Neurodevelopmental outcomes in children with congenital heart disease: evaluation and management: a scientific statement from the American Heart Association.* Circulation, 2012. **126**(9): p. 1143-72.
20. Wehby, G.L., et al., *Children with oral clefts are at greater risk for persistent low achievement in school than classmates.* Arch Dis Child, 2015. **100**(12): p. 1148-54.
21. Clausen, N.G., S. Kähler, and T.G. Hansen, *Systematic review of the neurocognitive outcomes used in studies of paediatric anaesthesia neurotoxicity.* Br J Anaesth, 2018. **120**(6): p. 1255-1273.
22. Boyd, P.A., et al., *Paper 1: The EUROCAT network—organization and processes†.* Birth Defects Res A Clin Mol Teratol, 2011. **91**(S1): p. S2-S15.
23. Greenlees, R., et al., *Paper 6: EUROCAT member registries: organization and activities.* Birth Defects Res A Clin Mol Teratol, 2011. **91 Suppl 1**: p. S51-s100.
24. Kinsner-Ovaskainen, A., et al., *A sustainable solution for the activities of the European network for surveillance of congenital anomalies: EUROCAT as part of the EU Platform on Rare Diseases Registration.* European Journal of Medical Genetics, 2018. **61**(9): p. 513-517.
25. Dolk, H., et al., *Detection and investigation of temporal clusters of congenital anomaly in Europe: seven years of experience of the EUROCAT surveillance system.* Eur J Epidemiol, 2015. **30**(11): p. 1153-1164.
26. Loane, M., et al., *Paper 4: EUROCAT statistical monitoring: Identification and investigation of ten year trends of congenital anomalies in Europe.* Birth Defects Res A Clin Mol Teratol, 2011. **91**(S1): p. S31-S43.
27. Salemi, J.L., et al., *The Accuracy of Hospital Discharge Diagnosis Codes for Major Birth Defects: Evaluation of a Statewide Registry With Passive Case Ascertainment.* J Public Health Manag Pract, 2016. **22**(3): p. E9-e19.
28. Wang, Y., P.K. Cross, and C.M. Druschel, *Hospital discharge data: can it serve as the sole source of case ascertainment for population-based birth defects surveillance programs?* J Public Health Manag Pract, 2010. **16**(3): p. 245-51.
29. Andrade, S.E., et al., *Validity of health plan and birth certificate data for pregnancy research.* Pharmacoepidemiol Drug Saf, 2013. **22**(1): p. 7-15.
30. Blais, L., et al., *Validity of congenital malformation diagnostic codes recorded in Québec's administrative databases.* Pharmacoepidemiol Drug Saf, 2013. **22**(8): p. 881-9.

For peer review only

31. Steele, A., et al., *A quality assessment of reporting sources for microcephaly in Utah, 2003 to 2013.* Birth Defects Res A Clin Mol Teratol, 2016. **106**(11): p. 983-988.
32. EUROCAT. *Guide 1.4*. 14/02/2018]; Available from: http://www.eurocat-network.eu/aboutus/datacollection/guidelinesforregistration/guide1_4.
33. Garne, E., et al., *Paper 5: Surveillance of multiple congenital anomalies: Implementation of a computer algorithm in European registers for classification of cases.* Birth Defects Res A Clin Mol Teratol, 2011. **91**(S1): p. S44-S50.
34. Herskind, A.M., et al., *Comparison of Late Mortality Among Twins Versus Singletons With Congenital Heart Defects.* The American Journal of Cardiology, 2017. **119**(10): p. 1680-1686.
35. Gnanendran, L., et al., *Neurodevelopmental outcomes of preterm singletons, twins and higher-order gestations: a population-based cohort study.* Arch Dis Child Fetal Neonatal Ed, 2015. **100**(2): p. F106-F114.
36. Wang, W., et al., *Outcomes of Congenital Diaphragmatic Hernia in One of the Twins.* Am J Perinatol, 2019. **36**(12): p. 1304-1309.
37. Martinka, D., et al., *Respiratory morbidity in late preterm twin infants.* Arch Gynecol Obstet, 2019. **300**(2): p. 337-345.

Author contributions

JKM is the principal author and the project's Scientific Coordinator; ML is the Data Coordinator; EG is the Clinical Coordinator; JD, AN, AP, JR and HdW are Steering Committee and Standardisation Committee members with JKM, ML and EG; IB and ALB are Steering Committee members; AR is a Standardisation Committee member; JT is the senior statistician; JG the research fellow; HC the project manager. All authors contributed to, read and approved the final manuscript.

Collaborators

All below named members of the EUROlinkCAT Consortium have contributed to the development of the protocol with their knowledge and experience of the surveillance of congenital anomalies. Names are given in Consortium organisation numerical order:

Population Health Research Institute, St George's, University of London, UK: Abigail Reid, Liz Limb, Gillian Briggs, Nicholas Connor

Faculty of Life and Health Sciences, Ulster University, Northern Ireland, UK: Joanne Given

Hospital Lillebaelt, Region Syddanmark, Denmark and Section of Epidemiology, University of Copenhagen, Denmark: Stine Kjaer Urhoj, Mads Damkjær, Christina Neergaard Pedersen

Population Health Sciences Institute, Faculty of Medical Sciences, Newcastle University, Newcastle upon Tyne, UK: Svetlana V. Glinianaia

Emila Romagna Registry of Birth Defects (IMER), University of Ferrara, Italy: Gianni Astolfi, Annarita Armaroli; Neonatal Intensive Care Unit, IMER Registry, Department of Medical Sciences, University Hospital of Ferrara, Italy: Elia Ballardini; Territorial Care Service - Emilia Romagna Health Authority, Italy: Aurora Puccini

Klinika za dječje bolesti Zagreb, Croatia: Ljubica Boban

National Research Council-Institute of Clinical Physiology (Consiglio Nazionale delle Ricerche-Istituto di Fisiologia Clinica), Pisa, Italy: Michele Santoro, Alessio Coi, Silvia Baldacci, Lorena Mezzasalma

For peer review only

University of Groningen, University Medical Center Groningen, Department of Genetics, Groningen, the Netherlands: L. Renée Lutke, Nicole H.A. Siemensma-Mühlenberg
Public Health Wales National Health Service Trust, Wales, UK: David Tucker
Institut National de la Santé et de la Recherche Médicale, Paris, France: Babak Khoshnood, Nathalie Bertille, Nathalie Lelong, Makan Rahshenas
Rare Diseases Research Unit, Foundation for the Promotion of Health and Biomedical Research in the Valencian Region, Valencia, Spain; Public Health and Preventive Medicine Department, University of València, Spain; Public Health Regional Health Administration (DG Salud Publica y Adicciones), Generalitat Valenciana, Valencia, Spain: Clara Cavero Carbonell, Óscar Zurriaga, Laia Barrachina Bonet, Laura García Villodre
Uniwersytet Medyczny im. Karola Marcinkowskiego w Poznaniu, Poznań, Poland: Anna Jamry-Dziurla
Finnish Institute for Health and Welfare, Finland: Anna Heino, Sonja Kiuru-Kuhlefelt, Mika Gissler
OMNI-Net for Children International Charitable Fund, Rivne, Ukraine: Wladimir Wertelecki, Lyubov Yevtushok, Nataliia Zymak-Zakutnia, Diana Akhmedzhanova, Lyubov Ostapchuk, Oksana Tsizh, Serhii Lapchenko
Epidemiology Department, National Registry of Congenital Anomalies, National Institute of Health Doctor Ricardo Jorge: Carlos Matias Dias, Ausenda Machado, Ana João Santos, Liliana Antunes, Paula Braz
Register of Congenital Malformations, Centre Hospitalier Universitaire de La Réunion, Île de la Réunion, France: Hanitra Randrianaivo-Ranjatoelina, Benedicte Bertaut Nativel
Provinciaal Instituut voor Hygiëne (PIH), Antwerp, Belgium: Vera Nelen, Guy Thys, Elly Den Hond
Departamento de Salud Gobierno Vasco, Basque Country, Spain: Olatz Mokoroa Carollo
Swansea University, Wales, UK: Daniel Thayer, Ieuan Scanlon, Sue Jordan
Department of Global Public Health and Primary Care, University of Bergen, Bergen, Norway and Division of Mental and Physical Health Norwegian Institute of Public Health, Bergen, Norway: Kari Klungsøyr
Directorate for Health Information and Research, Malta: Miriam Gatt

Funding statement

This project has received funding from the European Union's Horizon 2020 Research and Innovation programme under grant agreement No 733001 for 5 years: 1st January 2017 to 31st December 2021.

Data statement

The standardised methods and common data models will all be available freely on the EUROlinkCAT website. Requests for collaboration to analyse data from the study dataset are encouraged and should be addressed to the EUROlinkCAT Management Team, by contacting JKM. Interactive results tables will be accessible on the EUROlinkCAT website (https://www.eurolinkcat.eu/) later in the study.

Competing interests statement

The authors declare that they have no competing interests.

Figure legends

Figure 1: Structure of mortality and EUROCAT data used for analyzing children's survival

Figure 2: Structure of hospital admissions, prescription data and EUROCAT data used for analyzing children's morbidity

For peer review only

Patient data - 1 row per EUROCAT case							
Child ID	Child date of Birth	Child date of death	Type Matching	Variables relating to linkage	Variables relating to child	Variables relating to mortality	Variables relating to mother

Figure 1: Structure of mortality and EUROCAT data used for analysing children's survival

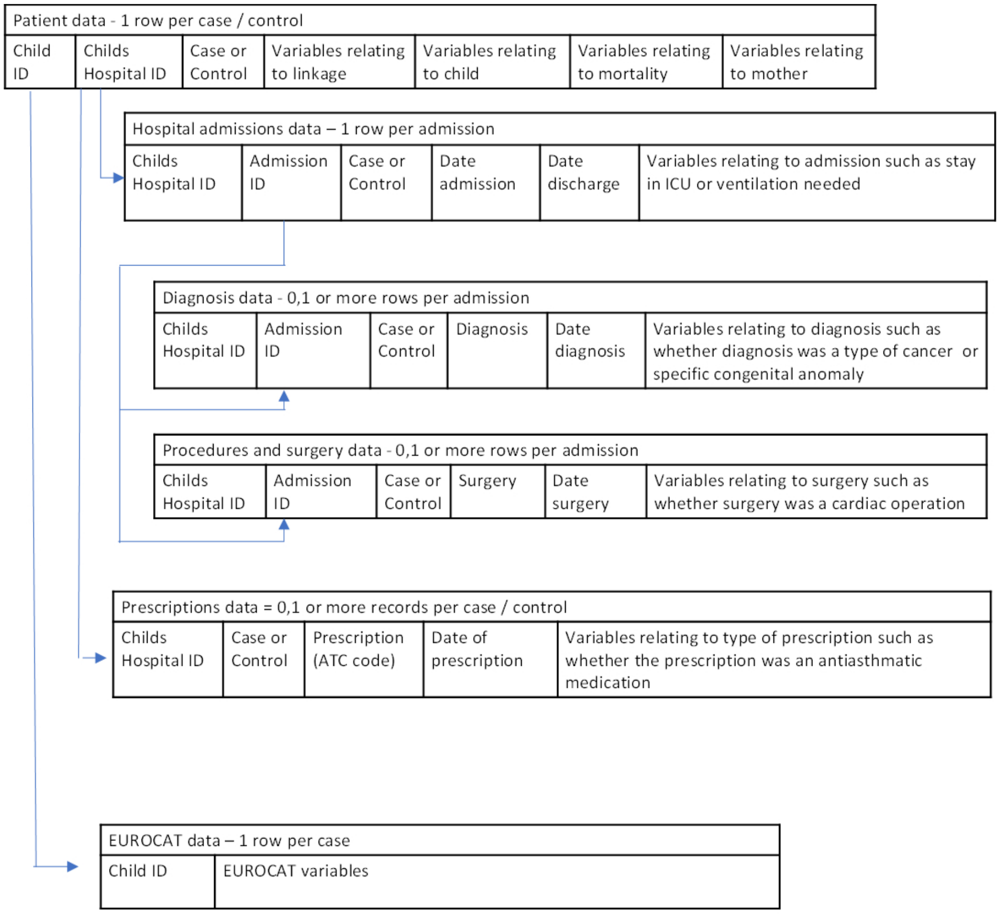

Figure 2: Structure of hospital admissions, prescription data and EUROCAT data used for analysing children's morbidity

Associated with document Ref. Ares(2016)3899547 - 25/07/2016

Proposal Evaluation Form

European Commission	**EUROPEAN COMMISSION**<br>Horizon 2020 - Research and Innovation Framework Programme	**Evaluation Summary Report - Research and innovation actions/Innovation actions**

Call: H2020-SC1-2016-RTD
Funding scheme: RIA
Proposal number: 733001
Proposal acronym: EUROlinkCAT
Duration (months): 60
Proposal title: EUROlinkCAT: Establishing a linked European Cohort of Children with Congenital Anomalies
Activity: SC1-PM-04-2016

N.	Proposer name	Country	Total Cost	%	Grant Requested	%
1	QUEEN MARY UNIVERSITY OF LONDON	UK	2,356,938	32.08%	2,356,938	32.08%
2	UNIVERSITY OF ULSTER	UK	1,402,544	19.09%	1,402,544	19.09%
3	REGION SYDDANMARK	DK	642,878	8.75%	642,878	8.75%
4	UNIVERSITY OF NEWCASTLE UPON TYNE	UK	558,875	7.61%	558,875	7.61%
5	UNIVERSITA DEGLI STUDI DI FERRARA	IT	343,218	4.67%	343,218	4.67%
6	Klinika za dječje bolesti Zagreb	HR	144,100	1.96%	144,100	1.96%
7	CONSIGLIO NAZIONALE DELLE RICERCHE	IT	200,462	2.73%	200,462	2.73%
8	ACADEMISCH ZIEKENHUIS GRONINGEN	NL	319,014	4.34%	319,014	4.34%
9	PUBLIC HEALTH WALES NATIONAL HEALTH SERVICE TRUST	UK	135,732	1.85%	135,732	1.85%
10	INSTITUT NATIONAL DE LA SANTE ET DE LA RECHERCHE MEDICALE (INSERM)	FR	26,282	0.36%	26,282	0.36%
11	FUNDACION PARA EL FOMENTO DE LA INVESTIGACION SANITARIA Y BIOMEDICA DELA COMUNITAT VALENCIANA	ES	116,838	1.59%	116,838	1.59%
12	UNIWERSYTET MEDYCZNY IM KAROLA MARCINKOWSKIEGO W POZNANIU	PL	138,775	1.89%	138,775	1.89%
13	TERVEYDEN JA HYVINVOINNIN LAITOS	FI	340,195	4.63%	340,195	4.63%
14	International Charitable Fund "OMNI-Net for Children"	UA	42,495	0.58%	42,495	0.58%
15	OTTO-VON-GUERICKE-UNIVERSITAET MAGDEBURG	DE	52,786	0.72%	52,786	0.72%
16	INSTITUTO NACIONAL DE SAUDE DR. RICARDO JORGE	PT	98,328	1.34%	98,328	1.34%
17	Centre Hospitalier Universitaire de La Réunion	FR	68,662	0.93%	68,662	0.93%
18	APB PIH	BE	50,582	0.69%	50,582	0.69%
19	ASOCIACION INSTITUTO BIODONOSTIA	ES	88,908	1.21%	88,908	1.21%
20	Biomedical Computing Limited	UK	138,072	1.88%	138,072	1.88%
21	Redburn Solutions Limited	UK	82,388	1.12%	82,388	1.12%
	Total:		7,348,073		7,348,073	

Abstract:
Over 130,000 children born in Europe every year will have a congenital anomaly (CA; birth defect). These CAs, which are often rare diseases, are a major cause of infant mortality, childhood morbidity and long-term disability. EUROCAT is an established European network of population-based registries for the epidemiologic surveillance of CAs. EUROlinkCAT will use the EUROCAT infrastructure to support 21 EUROCAT registries in 13 European countries to link their CA data to mortality, hospital discharge, prescription and educational databases. Each registry will send standard aggregate tables and analysis results to a Central Results Repository (CRR) thus respecting data security issues surrounding sensitive data. The CRR will contain standardised summary data and analyses on an estimated 200,000 children with a CA born from 1995 to 2014 up to age 10, enabling hypotheses on their health and education to be investigated at an EU level. This enhanced information will allow optimisation of personalised care and treatment decisions for children with rare CAs. Registries will be supported in using social media platforms to connect with families who live with CAs in their regions. A novel sustainable e-forum, “ConnectEpeople”, will link these families with local, national and international registries and information resources. ConnectEpeople will involve these families in setting research priorities and ensuring a meaningful dissemination of results. Findings will provide evidence to inform national treatment guidelines, such as concerning screening programs, to optimise diagnosis, prevention and treatment for these children and reduce health inequalities in Europe. An economic evaluation of the hospitalisation costs associated with CA will be provided The CRR and associated documentation, including linkage and standardisation procedures and “ConnectEpeople” forum will be available post-EUROlinkCAT thus facilitating future local and EU level analyses.

Evaluation Summary Report

Evaluation Result

Total score: 15.00 (Threshold: 12)

Form information

SCORING

Scores must be in the range 0-5.

Interpretation of the score:

For peer review only - http://bmjopen.bmj.com/site/about/guidelines.xhtml

*0 The **proposal fails to address the criterion** or cannot be assessed due to missing or incomplete information.*

***1 Poor.** The criterion is inadequately addressed, or there are serious inherent weaknesses.*

***2 Fair.** The proposal broadly addresses the criterion, but there are significant weaknesses.*

***3 Good.** The proposal addresses the criterion well, but a number of shortcomings are present.*

***4 Very good.** The proposal addresses the criterion very well, but a small number of shortcomings are present.*

***5 Excellent.** The proposal successfully addresses all relevant aspects of the criterion.Any shortcomings are minor.*

Criterion 1 - Excellence

Score: __5.00__ (Threshold: 4/5.00 , Weight: -)

The following aspects will be taken into account, to the extent that the proposed work corresponds to the topic description in the work programme:
Clarity and pertinence of the objectives
Soundness of the concept, and credibility of the proposed methodology
Extent that proposed work is beyond the state of the art, and demonstrates innovation potential (e.g. ground-breaking objectives, novel concepts and approaches, new products, services or business and organisational models)
Appropriate consideration of interdisciplinary approaches and, where relevant, use of stakeholder knowledge

EUROlinkCAT will exploit the European network of congenital anomaly registry (EUROCAT) infrastructure, seeking to link the data from these 21 registries (from 13 countries) to mortality, morbidity, hospital discharge, prescription and educational databases. This is a highly compelling application from an established network of institutions and investigators and the project builds on previous experience on data integration and harmonisation.

The concept of the proposed study is original and clear, and the proposed work is ambitious and achievable.

The proposal is timely; currently, limited information is available, especially in Europe, on the survival, health determinants, clinical course, hospitalisation costs, and educational achievements of children with congenital anomalies. The data on a very large number of children with congenital anomalies are impressive, with sizeable populations for rare disorders.

The project presents a unique opportunity to fill an important gap. Several data sources will be linked in several European countries to understand better the outcome and other aspects of congenital anomalies, which will inform families, health professionals and policy makers. The "ConnectEpeople" forum concept is novel, seeking more direct engagement with the parents of children with congenital anomalies.

The project plans to identify best practices for treatment options, evaluate the accuracy of existing electronic health care databases, and engage the relevant health authorities by establishing an Action Advisory Panel.

The proposal utilizes the appropriate interdisciplinary approaches and incorporates stakeholders' knowledge to understand better and improve clinical practice and health policy.

The potential problems with some of the data sources are not fully addressed.

Criterion 2 - Impact

Score: __5.00__ (Threshold: 4/5.00 , Weight: -)

The following aspects will be taken into account:
The extent to which the outputs of the project would contribute to each of the expected impacts mentioned in the work programme under the relevant topic
Any substantial impacts not mentioned in the work programme, that would enhance innovation capacity, create new market opportunities, strengthen competitiveness and growth of companies, address issues related to climate change or the environment, or bring other important benefits for society
Quality of the proposed measures to:
- exploit and disseminate the project results (including management of IPR), and to manage research data where relevant
- communicate the project activities to different target audiences

The expected impacts including conceptual, methodological, analytical impacts of the project are well described. The project results will help inform policy and contribute to formulating best practice guidelines and improve clinical practice. The project will enable parents and patients to access pertinent information. The concept is well aligned with the impacts listed in the work programme.

The project can bring important benefits for society (evidence-based information, resource allocation, best clinical practice, socioeconomic research, policy change). Future work and programmes/registries can also benefit from the recommendations on coding standardisation but the panellists felt that potential impacts could have been addressed more explicitly with additional considerations.

The dissemination component of the proposal is adequately addressed with engagement of several stakeholders.

Facilitated by the Action Advisory Panel, the communication of project activities and findings to various target audiences are well summarised and liaisons are nominated to collaborate with relevant other networks and organisations.

Criterion 3 - Quality and efficiency of the implementation

Score: __5.00__ (Threshold: 3/5.00 , Weight: -)

The following aspects will be taken into account:
Quality and effectiveness of the work plan, including extent to which the resources assigned to work packages are in line with their objectives and deliverables

For peer review only - http://bmjopen.bmj.com/site/about/guidelines.xhtml

Appropriateness of the management structures and procedures, including risk and innovation management
Complementarity of the participants and extent to which the consortium as a whole brings together the necessary expertise
Appropriateness of the allocation of tasks, ensuring that all participants have a valid role and adequate resources in the project to fulfil that role

The project presents a coherent and effective work plan. The work plan is well distributed in WPs, and the description, deliverables, and milestones are well described. An adequate Gannt chart is included with timing of the different WPs.

Management structure and procedures are appropriate. Innovation and risk management (with risk mitigation measures) are adequately explained.

The consortium brings together partners with strong and relevant experience and expertise. The various partners have a long lasting collaborative history and show strong publication records, complementing each other in several areas.

The allocation of tasks is well planned and the resources requested are compatible with the work to be performed and appropriately allocated to the participants.

Gender differences are well described and fully taken into account in the study.

Scope of the proposal

Status: **Yes**

Comments (in case the proposal is out of scope)

Not provided

Operational Capacity

Status: **Operational Capacity: Yes**

If No, please list the concerned partner(s), the reasons for the rejection, and the requested amount.

Not provided

Exceptional funding of third country participants/international organisations

A third country participant/international organisation not listed in General Annex A to the Main Work Programme may exceptionally receive funding if their participation is essential for carrying out the project (for instance due to outstanding expertise, access to unique know-how, access to research infrastructure, access to particular geographical environments, possibility to involve key partners in emerging markets, access to data, etc.). (For more information, see the Online Manual)

Based on the information provided in the proposal, we consider that the following participant(s)/international organisation(s) that requested funding should exceptionally be funded:
(Please list the Name and acronym of the applicant, Reasons for exceptional funding and the Requested grant amount.)

Not provided

Based on the information provided in the proposal, we consider that the following participant(s)/international organisation(s) that requested funding should NOT be funded:
(Please list the Name and acronym of the applicant, Reasons for exceptional funding and the Requested grant amount.)

Not provided

Use of human embryonic stem cells (hESC)

Status: **No**

If yes, please state whether the use of hESC is, or is not, in your opinion, necessary to achieve the scientific objectives of the proposal and the reasons why. Alternatively, please also state if it cannot be assessed whether the use of hESC is necessary or not because of a lack of information.

Not provided

For peer review only

European Commission

PARTICIPANT PORTAL DIGITAL SEAL

This document is digitally sealed. The digital sealing mechanism uniquely binds the document to the modules of the Participant Portal of the European Commission, to the transaction for which it was generated and ensures its integrity and authenticity.

Any attempt to modify the content will lead to a breach of the electronic seal, which can be verified at any time by clicking on the digital seal validation symbol.

Commission européenne/Europese Commissie, 1049 Bruxelles/Brussel, BELGIQUE/BELGIË - Tel. +32 22991111
